# *Onchocerca volvulus* infection prevalence and intensity in Logo and Nyarambe Health Zones in Ituri, Democratic Republic of the Congo in 2010 and in 2021–2023: results of screening for clinical trials of moxidectin versus ivermectin

**DOI:** 10.1186/s13071-025-07199-8

**Published:** 2026-03-16

**Authors:** Françoise N. Ngave, Deogratias U. Wonyarossi, Germain M. Abhafule, Joël L. Mande, Amos Nyathirombo, Claude B. Uvon, Anuarite A. Raciu, Michel Mandro, Pascal T. Adroba, Tony O. Ukety, Innocent A. Mananu, Gisèle L. Abeditho, Jules U. Upenjirwoth, Carine M. Aliang’o, Jean de Dieu N. Unega, Maurice M. Nigo, Didier Bakajika, Jean-Paul U. Uvoyo, Germain L. Mambandu, Christine M. Halleux, Michel Vaillant, Anna Schritz, Beatriz Mosqueira, Mupenzi Mumbere, Sally Kinrade, Annette C. Kuesel

**Affiliations:** 1 Centre de Recherche en Maladies Tropicales, Rethy, Democratic Republic of the Congo; 2 Division Provinciale de La Santé de L’Ituri (Ituri Provincial Health Division), Bunia, Democratic Republic of the Congo; 3 World Health Organization Special Programme for Research and Training in Tropical Diseases (TDR), Geneva, Switzerland; 4 Competence Center for Methodology and Statistics, Luxembourg Institute of Health, Strassen, Grand Duchy of Luxembourg; 5 Medicines Development for Global Health, Melbourne, Australia

**Keywords:** Onchocerciasis, Skin microfilariae prevalence, Moxidectin, Ivermectin, Northern Ituri, DRC

## Abstract

**Background:**

In Ituri Province, 7576 and 1056 volunteers living in Logo and Nyarambe Health Zone (HZ), respectively, were screened in 2021–2023 for two studies comparing moxidectin and ivermectin in individuals with ≥ 0 *Onchocerca volvulus* skin microfilariae density (SmfD, microfilariae/mg skin). Site selection was based on the trial capacity established for the moxidectin Phase 3 study and SmfD measured among 1373 and 36 individuals screened in HZ Logo and Nyarambe, respectively, in 2010. We compared the SmfD measured in 2010 and 2021–2023 in Logo HZ where ivermectin mass administration was never implemented and provide descriptive statistics for SmfD from Nyarambe HZ.

**Methods:**

Four skin snips from each consenting/assenting individual ≥ 12 years old were weighed and incubated in isotonic saline for ≥ 8 h. Emerged microfilariae were counted and SmfD calculated as the mean of the microfilariae/mg skin of each snip. Other data collected included age, gender, village of residence, and history of ivermectin treatment.

**Results:**

In 2010 and 2021–2023, respectively, adults (18–93 years old) represented 92.1% and 73.2%, and women 36.9% and 46.6% of the 1373 and 7547 volunteers from Logo HZ without reported prior ivermectin treatment. Among these adults and adolescents (12–17 years), no microfilariae were detected in snips from 23.3% and 26.9% in 2010 and 89.8% and 96.8% in 2021–2023, respectively, with mean ± standard deviation SmfD being 24.30 ± 35.52 and 11.8 ± 18.37 in 2010 and 1.1 ± 6.44 and 0.3 ± 2.62 in 2021–2023, respectively.

**Conclusions:**

Given that the reduction in infection prevalence and intensity in Logo HZ cannot be attributed to ivermectin distribution, it has to be due to reduction in infective vector biting rates, possibly linked to a recently proposed change in vector species triggered by land-use changes. Because SmfD reflects transmission events approximately 2–15 years earlier, infective vector biting rate assessment is needed to determine current transmission rates. Reduced transmission shifts macrofilariae age distribution toward older macrofilariae with lower reproductive capacity. Comparison of the results from the Phase 3 and the ongoing efficacy study might help determine whether drug susceptibility changes significantly with macrofilariae age. Should that be the case, transmission models evaluating the impact of mass drug administrations could be adjusted.

**Graphical Abstract:**

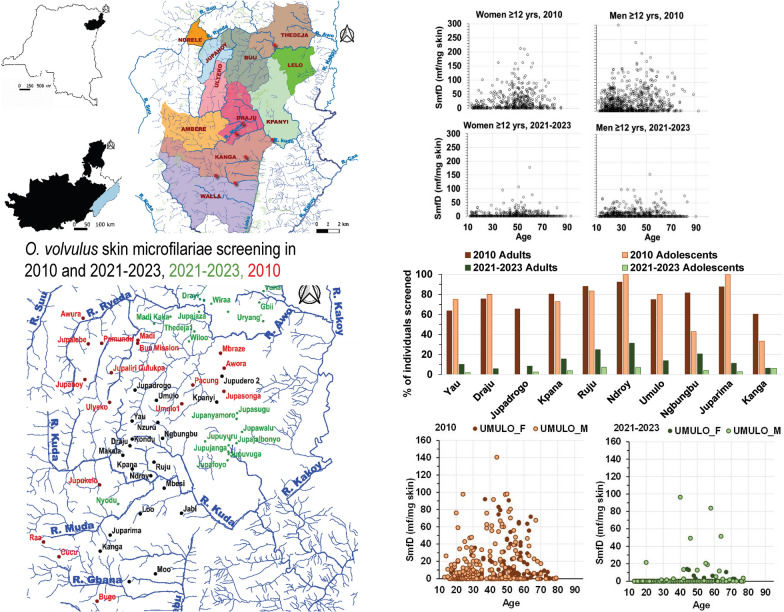

**Supplementary Information:**

The online version contains supplementary material available at 10.1186/s13071-025-07199-8.

## Background

Onchocerciasis, a *Simulium*-borne parasitic disease caused by *Onchocerca volvulus*, is targeted for elimination (interruption of parasite transmission). Current World Health Organization (WHO) 2030 targets are WHO-verified elimination in 12 countries, stop of mass drug administration of ivermectin (MDAi), the principal intervention strategy, in at least one focus in 34 countries, and > 16 countries having stopped MDAi for ≥ 50% and > 12 countries having stopped MDA for 100% of the population by 2030 [1].

In Colombia, Ecuador, Guatemala, and Mexico, the WHO verified elimination following ≥ 23 rounds of biannual MDAi, complemented in around 300 communities by quarterly MDAi. This reduced the number of people requiring interventions in the Americas from 0.538 million to around 0.039 million people living in the endemic area spanning the Brazil–Venezuela border in 2023 [2–7].

More than 90% of people infected or at risk of infection live in sub-Saharan Africa (SSA), where many endemic areas are very large and hyperendemic [8–11]. For these and other reasons, elimination across SSA is most challenging [12]. The initial objective of the African Programme for Onchocerciasis Control (APOC, 1995–2015 [13]) was to establish effective and self-sustainable community-based ivermectin treatment throughout the endemic areas within its geographical scope (central, eastern, and southern SSA and Liberia) and, if possible, to eliminate the vector and hence the disease by using environmentally safe methods in selected foci to “ultimately realize the goal of elimination of onchocerciasis as a disease of public health and socio-economic importance throughout Africa” [14, 15]. APOC addressed its first objective through working with countries to establish community-directed treatment with ivermectin (CDTI) [16, 17] in meso- and hyperendemic areas, i.e., where rapid epidemiological assessment (REA) [18, 19] estimated that > 20% or > 40% of adults ≥ 20 years, respectively, had subcutaneous onchocercal nodules (nodule prevalence) [10, 11, 20]. After research [21, 22] and APOC-funded parasitological assessments [23, 24] suggested that interruption of parasite transmission may have or could soon be achieved in many areas under long-term annual CDTI, APOC’s objective expanded to include all endemic SSA countries and Sudan and to eliminate parasite transmission in some countries by 2020 and in 80% of countries by 2025 [25]. Following devolution of APOC responsibilities to countries or the WHO Expanded Special Project for Elimination of Neglected Tropical Diseases (WHO/ESPEN), the WHO revised these targets [1]. As of 2023, MDAi had been stopped for 17.8 million people across Equatorial Guinea, Ethiopia, Mali, Nigeria, Senegal, Togo, and Uganda, while MDAi continues for 247.8 million people in the WHO African Region. Across Sudan and Yemen in the WHO Eastern Mediterranean Region, ivermectin distribution has been discontinued for 0.51 million and continues for 1.7 million people [26].

APOC data reviews and expert consultations on the needs for interrupting parasite transmission across the countries within its mandate concluded that alternative treatment strategies are required in many areas [25, 27]. One strategy identified is use of moxidectin rather than ivermectin in selected areas.

Moxidectin development was initiated by the UNICEF/UNDP/World Bank/WHO Special Programme for Research and Training in Tropical Diseases (WHO/TDR) in the late 1990s in consultation and with support of the Onchocerciasis Control Programme in West Africa (OCP, 1974–2002) and APOC [28–39]. In 2014, WHO licensed all data at its disposal to the Australian not-for-profit health charity Medicines Development for Global Health (MDGH) to register moxidectin with a stringent regulatory authority and make moxidectin available to countries. In 2018, moxidectin was approved by the United States of America Food and Drug Administration (US-FDA) [40]. The pharmacokinetic and safety data from a pediatric study in Ghana [41] supported approval of moxidectin for ages  ≥ 4 years by the Ghana Food and Drugs Authority (G-FDA) [42–44] and the US-FDA [45].

To provide the WHO and countries with additional data to decide on moxidectin’s inclusion in guidelines and policies, a randomized, double-blind, single-dose study (MDGH-MOX-3002) in individuals ≥ 4 years old with ≥ 0 skin microfilariae (mf) density (SmfD, mf/mg skin) obtained safety data in 5550 adults, 2289 adolescents (12–17 years), and 187 children (4–11 years) in the Democratic Republic of the Congo (DRC) randomized 4:1 to moxidectin:ivermectin and in 3240 adults, 890 adolescents, and 840 children in Côte d’Ivoire randomized 4:1 to moxidectin:ivermectin with concomitant administration of 400 mg albendazole. A randomized, double-blind, ivermectin-controlled study (MDGH-MOX-3001) including 323 participants ≥ 12 years old with ≥ 10 SmfD assessing the efficacy and safety of three annual and five biannual treatments is ongoing in DRC (protocols: https://mox4oncho-multimox.net/resources).

**Fig. 1:**
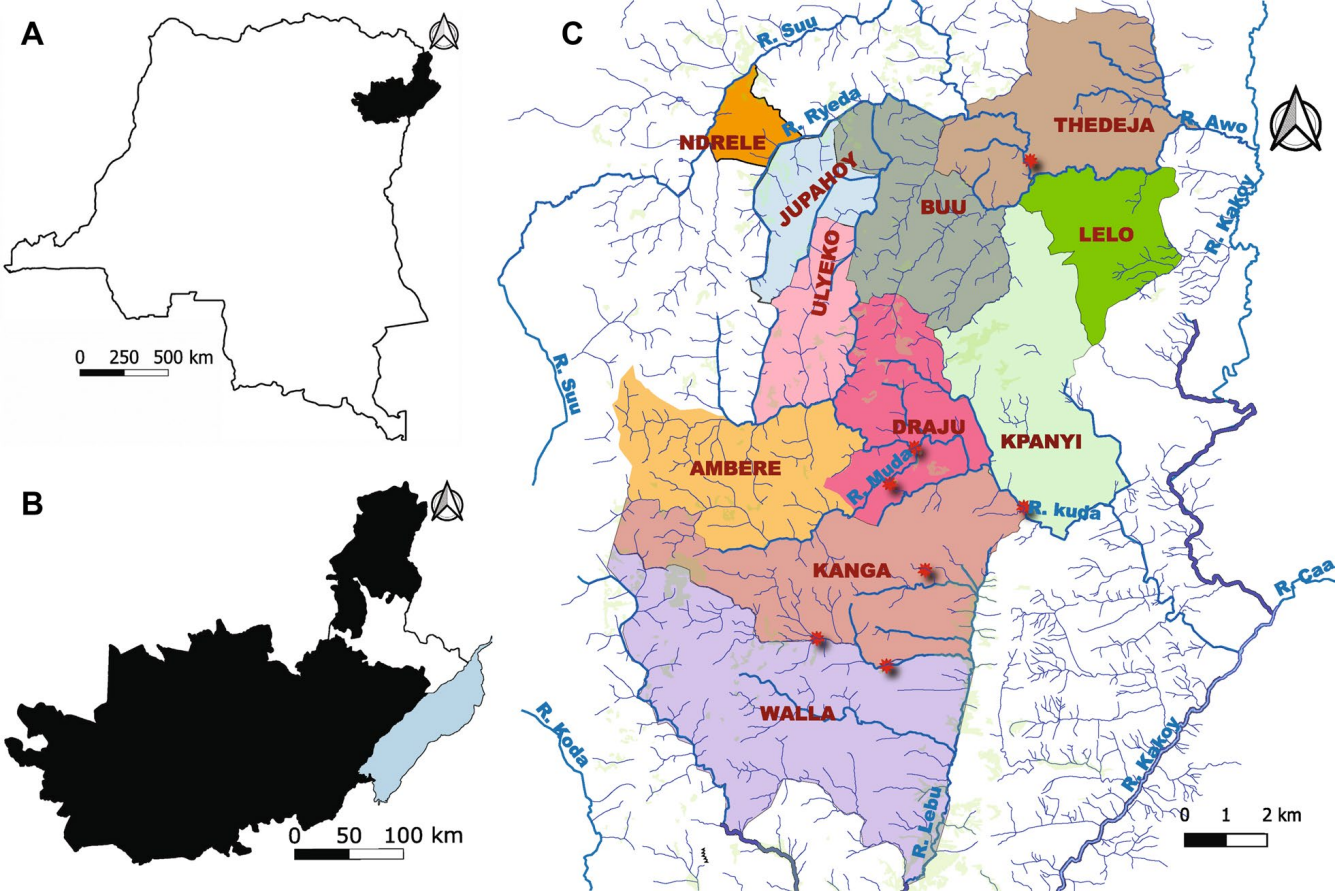
Location of study area within DRC. **A** Ituri Province (black) in the north east of DRC, **B** Mahagi territory (white) bordering Lake Albert (blue) within Ituri Province, **C** Health Areas where individuals were screened. Lelo and Kpanyi are in the Health Zone Nyarambe, all others in the Health Zone Logo. Red circles show places where villagers screened in 2021–2023 indicated blackfly nuisance

The majority of the efficacy and safety data supporting the regulatory approvals came from a Phase 3 (P3) study in Liberia, Ghana, and DRC [29, 37, 38]. One of the DRC study areas was in Northern Ituri (Fig. 1), where participants for studies MDGH-MOX-3002 and MDGH-MOX-3001 were recruited.

Here, we compare the SmfD obtained during screening for these studies in 2021–2023 and the P3 study in 2010 in ≥ 12 years old individuals in the Health Areas (Aires de Santé) that are part of the Health Zone (Zone de Santé) Logo and report the SmfD obtained in the Health Areas which are part of the Health Zone Nyarambe.

## Methods

### Trial registration

All studies were registered in Clinicaltrials.gov: the P3 study on 14 November 2008 (ID: NCT00790998), MDGH-MOX-3001 on 15 March 2019 (ID NCT03876262), and MDGH-MOX-3002 on 17 March 2020 (ID NCT04311671). Studies MDGH-MOX-3001 and MDGH-MOX-3002 are also registered in the Pan African Clinical Trials Registry (IDs PACTR202004639229710 and PACTR202003567524647).

### Regulatory agency and ethics committee approval

MDGH-MOX-3001 and MDGH-MOX-3002 protocols, information documents for potential participants, consent and assent forms, and study conduct were approved by the DRC National Ethics Committee (Comité National d’Ethique de la Santé) and the National Regulatory Agency (Direction de la Pharmacie et du Médicament/Autorité Congolaise de Règlementation Pharmaceutique, Ministère de la Santé Publique, Hygiène et Prévention (MoH)). Approvals for the P3 study were obtained from the Ethics Committee of the Ecole de la Santé Publique Université de Kinshasa and the MoH [29, 37, 38]. All documents were also approved by the WHO Ethics Review Committee.

### Recruitment and informed consent/assent with parental consent

Informed consent or assent with parental consent (IC/IA) was obtained in or close to the villages where volunteers lived and documented through signature or thumbprint in the presence of an independent literate witness.

Screening of volunteers ≥ 12 years old for the P3 study in Northern Ituri took place between January 2010 and January 2011 (referred to as “2010” since only 14/1409 volunteers were screened in 2011).

**Fig. 2:**
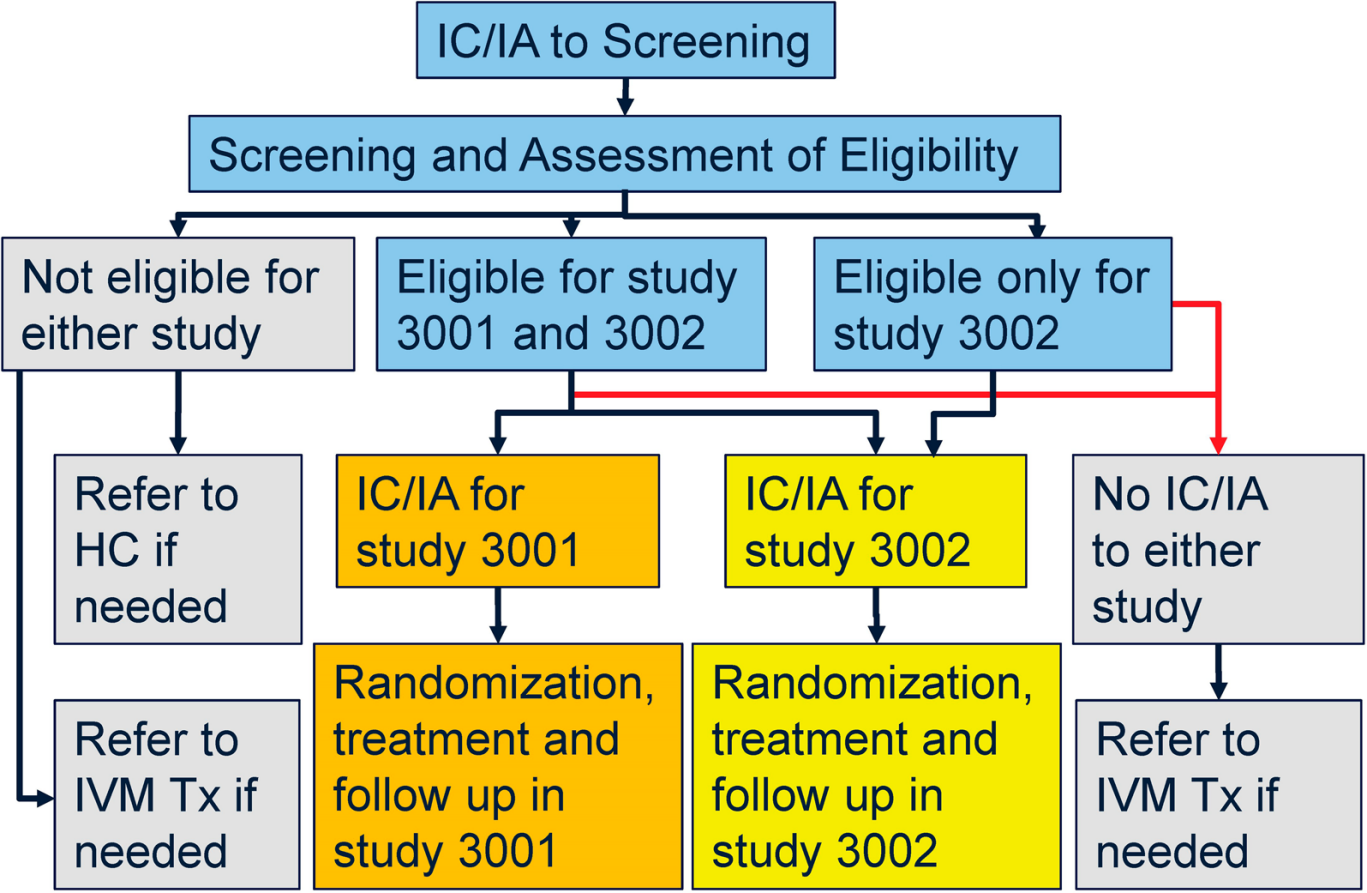
Schematics of nested recruitment for studies MDGH-MOX-3001 and MDGH-3002 in DRC. 3001, study MDGH-MOX-3001; 3002, study MDGH-MOX-3002; HC, Health Center; IC/IA, informed consent or assent with parental/guardian consent; IVM, ivermectin; Tx, treatment. Eligibility for MDGH-MOX-3001 required skin microfilariae density ≥ 10 mf/mg skin

“Nested recruitment” of volunteers ≥ 12 years old for MDGH-MOX-3001 and MDGH-MOX-3002 occurred from May 2021 to July 2023 (Fig. 2): (1) volunteers provided IC/IA to be screened based on information about screening procedures and their possible choice(s) based on the screening results; (2) during the discussion of these results, additional details about the studies were provided as indicated by each individual’s eligibility to inform IC/IA to study participation.

### Quantitation of skin microfilariae densities

Four skin snips (one from each iliac crest and calf) were taken with a 2-mm corneoscleral punch. Each snip was weighed and individually incubated in a well of a flat-bottom 96-well microtiter plate for ≥ 8 h in isotonic saline. The emerged microfilariae in each well were counted with an inverted microscope [28, 37]. Given that no mf were detected in the skin snips of around 97% of adolescents screened in 2021–2023, the MDGH-MOX-3002 protocol amendment to include 4–11-year-old children excluded SmfD measurement in children.

SmfD was calculated as the mean of the unrounded mf/mg skin of each snip. Thus, each individual with ≥ 1 mf detected across all snips is considered mf-positive. This is a more stringent criterion for mf-positivity than we used in our previous overview of the P3 study screening data [29].

### Demographic characteristics, village names, and GPS coordinates

For studies MDGH-MOX-3001 and MDGH-MOX-3002, age, gender, village, and Health Area of residence (as per the Division Provinciale de la Santé Ituri names and delineations introduced in 2014) were entered into the database for all screened.

The P3 study database included only study participants. The data for screen failures were extracted from the source documents. For this analysis, the village names reported in 2010 [29] were mapped to those introduced in 2014.

The village GPS coordinates are provided in Additional File 1 Table S1.

### Maps

Maps were generated with QGIS version 3.40.3. Shapefiles for DRC (ADM0) and Ituri Province (ADM1) were those available at the WHO. While the WHO has shapefiles for Health Zones (ADM2, not used in the maps), the WHO does not have shapefiles for Mahagi Territory and the Health Areas. These were obtained from the DRC MoH (COD2 and COD4, respectively). The Health Area shapes were corrected as necessary using QUICKOSM background by D.U.W. in collaboration with local healthcare staff.

### Study area

#### Location

Individuals screened lived in 9/26 Health Areas composing the Health Zone Logo and 2/22 Health Areas composing the Health Zone Nyarambe in the Mahagi Territory in the Ituri Province of DRC (Fig. 1) close to Lake Albert. While these Health Areas represent only a fraction of the Health Areas in each Health Zone, for convenience the Health Zone name is used below when referring to data across these nine and two Health Areas.

Additional File 1 Fig. S1 shows Fig. 1 C with 2021–2023 dwellings indicated. Additional File 1 Fig. S2 provides an overview of the study area, including the location of the Centre de Recherche en Maladies Tropicales de l’Ituri (CRMT) at the Hôpital Général de Réference Rethy and of villages included in 2002 and 2015 evaluations of *O. volvulus* infection prevalence. The area is mountainous, with people living primarily on the slopes or tops of lower hills and cultivating fields in the valleys close to rivers and streams (Additional File 1 Fig. S2 and Fig. S4).

#### River basins and villages

**Fig. 3:**
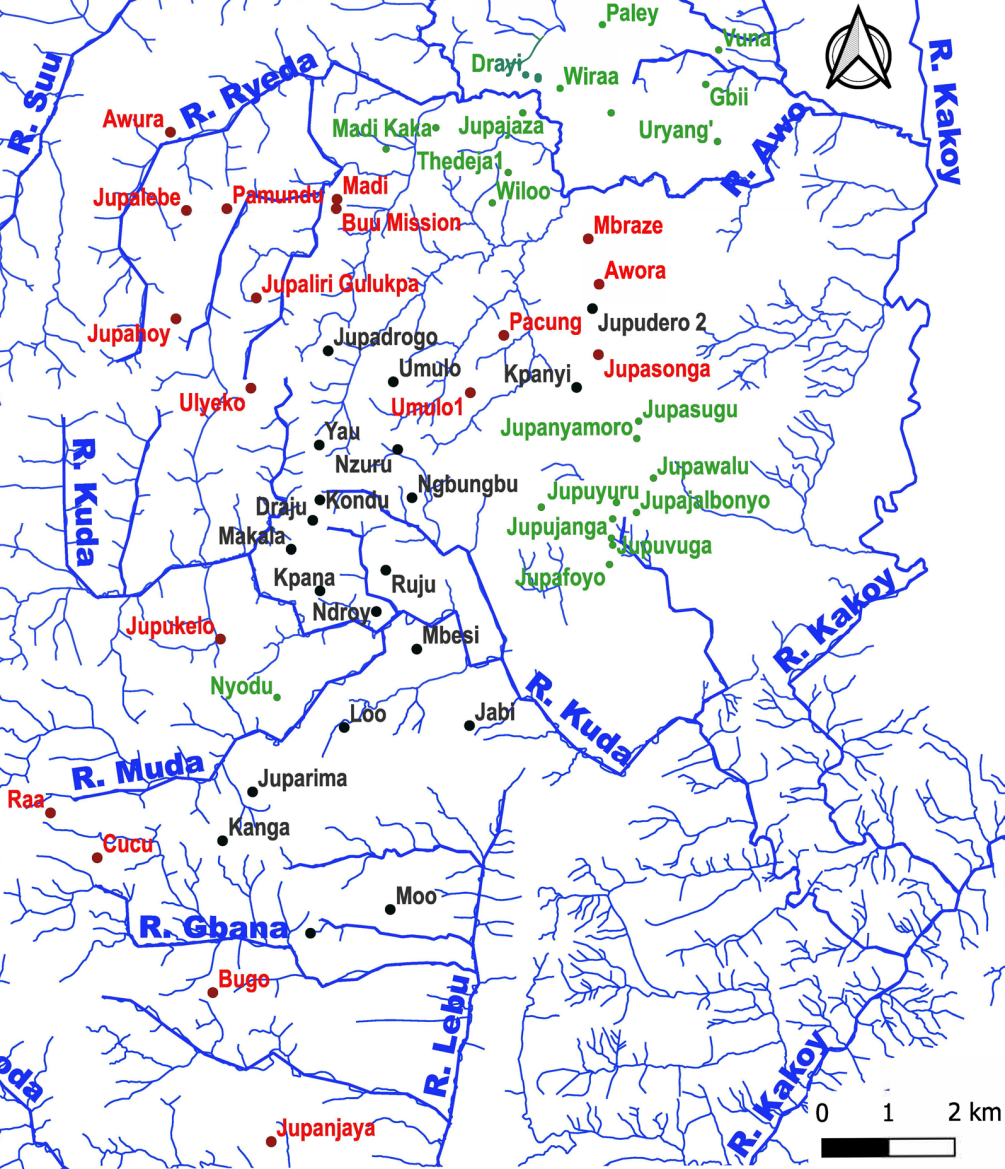
Villages of residence of individuals screened in 2010 and in 2021–2023. Villages where volunteers were screened in 2010 and 2021–2023 (black), only in 2021–2023 (green), and only in 2010 (red). Owing to the vicinity of many villages to each other, not all village names can be displayed. All villages and their GPS coordinates are included in Additional File 1 Table S1

On the basis of the topographical characteristics, the rivers, and their affluents, villages were assigned to three river basins: Awo in the North, Kuda in the center, and Lebu in the south (Fig. 3, Additional File 1 Fig. S2).

#### Results of 2002 rapid epidemiological assessment

In 2002, an APOC-funded REA conducted by co-authors T.O.U. and D.U.W. at the Projet Ophtalmologie à Nyankunde identified meso- and hyperendemic villages (Additional File 1 Table S2 and Fig. S4), which led to the area being designated as a “high risk”/priority area for CDTI implementation [10, 11].

The 2007 WHO/TDR decision to include this area in the P3 study was based on the REA results, the fact that CDTI had not been implemented, and consultation with APOC as well as the MoH. WHO/TDR investments into the necessary personnel capacity and infrastructure resulted in establishment of the CRMT (Additional File 1 Fig. S2). The CRMT capacity, the P3 screening data, and the fact that CDTI had not yet been implemented in the Health Zone Logo led to the selection of this area for studies MDGH-MOX-3001 and MDGH-MOX-3002. Additional File 1 Table S3 provides an overview of CRMT capacities, research experience, and diseases prevalent in the area (Additional File 1 Fig. S2).

#### Vectors

The vectors obtained through collection of aquatic stages and human landing catches in 2015–2018 were identified as *Simulium dentulosum* and *Simulium vorax* [46].

#### Socioeconomic characteristics

The majority of the population is poor with agricultural livelihoods, including subsistence farming. Production of coffee, mostly sold in Uganda, constitutes the main source of income and employment. The fields that people cultivate may not be close to or in the same river basin as their village of residence, and people from different villages may have fields close to each other. Men and women have different roles: men clear the fields and prepare water courses for watering and drainage, as required, while both men and women plow, sow, weed, and harvest. Field work occurs throughout the year given that the fertility of the area allows several harvests per year.

#### Ivermectin treatment history

The Health Zones Logo, Nyarambe, and Rethy were included in the agreement between WHO and the MoH that established the APOC CDTI-Project Ituri Nord, initiated in 2007 [47–49]. The 2007–2012 CDTI-Project Ituri Nord reports to APOC characterize the Health Zones Logo and Nyarambe as not requiring CDTI and did not cover the Health Zone Rethy [50, 51]. The Health Zone Rethy was included in the APOC CDTI Project Ituri Sud, initiated in 2012 [52]. As per the ESPEN Implementation Unit level onchocerciasis database (accessed 16 February 2025), CDTI was not implemented in the Health Zone Logo. For control of lymphatic filariasis (LF) in the Health Zone Nyarambe, between 181,468 and 225,278 people received ivermectin + albendazole annually in 2016–2019 and 2021–2022 (treatment coverage 80–84%), and in 2023, 197,640 people received ivermectin alone (treatment coverage 84%).

Health zones are areas delineated by the health system administration, not on the basis of criteria delineating an *O. volvulus* transmission zone [53–55]. Consequently, in the absence of any relevant data, it is unknown whether all or parts of Logo and Nyarambe Health Zones belong to the same or different transmission zones. The different ivermectin treatment history of the Health Zones Logo and Nyarambe and its possible impact on SmfD of those screened in 2021–2023 and parasite transmission is one of the reasons for data presentation by Health Zones.

### Ivermectin treatment history of individuals screened

None of the volunteers screened in 2010 had received ivermectin previously. In the P3 study, 472 volunteers (SmfD 10–299) received a single dose of 8 mg moxidectin (*n* = 315, geometric mean SmfD: pre-treatment 37.4, 12 months post-treatment 0.7) or 150 µg/kg ivermectin (*n* = 157, geometric mean SmfD: pre-treatment 40.7, 12 months post-treatment 8.0) in 2010 to January 2011 [29].

In 2021–2023, ivermectin treatment between 2 and 1800 days before screening was reported by 29/7576 (0.4%) volunteers from the Health Zone Logo and 9/1056 (0.9%) volunteers from the Health Zone Nyarambe, respectively. Volunteers were not asked about participation in the P3 study. Reliable post hoc identification of P3 participants was not possible. Our best estimate suggests that around 48 moxidectin- and 26 ivermectin-treated P3 participants were screened in 2021–2023. The treatment of any P3 participants with moxidectin or ivermectin cannot be considered as “prior ivermectin treatment” affecting SmfD measured in 2021–2023 because: (1) given the *O. volvulus* reproductive life of 10 ± 3 years [56–59], very few of the macrofilariae present in P3 study participants at the time of their treatment can have been alive/reproductively active during screening in 2021–2023; (2) the duration of the embryostatic effect of a single ivermectin or moxidectin dose on SmfD shows considerable interindividual variability [29, 37, 60, 61]. While its maximum is unknown, it is extremely unlikely to be ≥ 10 years in even a small percentage of those treated, which would be tantamount to a single dose being permanently sterilizing (or life-span shortening/cidal). While there are no data to support or refute such a hypothesis for moxidectin, available data for ivermectin do not support such an effect [62].

For individuals without prior ivermectin treatment, the SmfD reflects the individual’s intensity of infection and across individuals the prevalence of infection among those evaluated (as detectable on the basis of the number of skin snips, incubation medium, and duration of incubation [63]). In contrast, and given the variable long-term effect of a single ivermectin (or moxidectin) dose on an individual’s SmfD [29, 37, 60, 61], this is not necessarily the case for people who have taken ivermectin relatively recently. Consequently, our analysis focuses on individuals who did not report prior ivermectin treatment during screening.

### Data analysis

As noted above, Health Zone and Health Area are administrative boundaries, not epidemiological/parasite transmission relevant boundaries (i.e., transmission zones). We decided, nevertheless, to analyze the data by Health Zone with a focus on the data from Health Zone Logo because of the potential impact of IVM treatment for control of lymphatic filariasis in the Health Zone Nyarambe on *O. volvulus* transmission and the fact that only 36 individuals residing in the Health Zone Nyarambe participated in screening in 2010. Given the potential value of the data from the Health Zone Nyarambe for decisions on strategies toward elimination, descriptive statistics are provided for the data from Health Zone Nyarambe (primarily in Additional File 1).

For some analyses, SmfD were categorized as either 0 versus > 0 or as follows: 0, > 0 to  < 5, ≥ 5 to  < 10, ≥ 10 to  < 20, ≥ 20 to  < 30, ≥ 30 to  < 40, ≥ 40 to  < 50, ≥ 50 to  < 60, ≥ 60 to  < 80, ≥ 80.

Logistic regression with prevalence of infection (yes/no) as the dependent binary variable and age and gender as independent variables was used to analyze the data from 2010 and from 2021–2023 separately to identify the extent to which age and gender impacted the risk of being infected in each period. For 2021–2023, prior ivermectin treatment was included as an additional independent variable. The data obtained from the 10 villages in the Health Zone Logo where at least 20 individuals had been screened in both 2010 and 2021–2023 were analyzed by logistic regression as described above and also using the period (2010 and 2021–2023) as interaction effect for age, gender, and village.

Inferential analyses were conducted with R (R Foundation for Statistical Computing, Vienna, Austria). All confidence intervals (CI) are 95%. Descriptive analysis and figures were generated in Excel 365.

## Results

### Demographic characteristics

Most volunteers screened in 2010 (1373/1409, 97.4%) and in 2021–2023 (7603/8632, 88.1%) lived in the Health Zone Logo. Adults in Health Zone Logo represented 92.1% and 73.2% and women 36.9% and 46.6% of volunteers screened in 2010 and 2021–2023, respectively (Table 1). The mean age of both adults and adolescents without prior IVM treatment was somewhat higher in 2010 than 2021–2023 (Table 1). In 2021–2023, the age distribution among adolescents was shifted toward younger ages compared with 2010, while in 2010 the percentage of adults between approximately 45 and 65 years of age was higher than in 2021–2023 (Additional File 1 Fig. S5).

**Table 1:** Demographic characteristics

			No prior IVM treatment										Prior IVM treatment				
			2010	2010	2010	2010	2010	2021–2023	2021–2023	2021–2023	2021–2023	2021–2023	2021–2023	2021–2023	2021–2023	2021–2023	2021–2023
Health Zone	Age category	Gender	*N*	Mean age	SD age	Min age	Max age	*N*	Mean age	SD age	Min age	Max age	*N*	Mean age	SD age	Min age	Max age
Logo			1373	40.1	16.92	12	93	7547	32.0	17.59	12	93	29	38.1	20.57	14	84
	Adults		1265	42.2	15.91	18	93	5517	38.4	16.44	18	93	25	41.7	19.88	18	84
	Adults	F	471	48.9	14.33	18	85	2519	41.1	16.62	18	87	6	50.5	15.32	21	62
	Adults	M	794	38.3	15.48	18	93	2998	36.2	15.96	18	93	19	38.9	20.68	18	84
	Ado		108	15.2	1.55	12	17	2030	14.6	1.39	12	17	4	15.5	1.29	14	17
	Ado	F	35	14.8	1.50	12	17	1006	14.5	1.37	12	17	2	16.5	0.71	16	17
	Ado	M	73	15.4	1.54	12	17	1024	14.7	1.40	12	17	2	14.5	0.71	14	15
Nyarambe			36	45.6	16.09	18	70	1047	30.3	18.14	12	91	9	45.6	19.05	19	79
	Adults		36	45.6	16.09	18	70	690	38.4	17.50	18	91	9	45.6	19.05	19	79
	Adults	F	18	55.1	10.77	32	70	270	43.9	17.20	18	91	4	60.5	15.33	46	79
	Adults	M	18	36.0	14.95	18	66	420	34.8	16.77	18	86	5	33.6	12.16	19	48
	Ado							357	14.6	1.21	12	17	0				
	Ado	F						173	14.6	1.12	13	17					
	Ado	M						184	14.7	1.28	12	17					

*Ado* adolescents, *IVM* ivermectin, *SD* standard deviation

### Nyarambe Health Zone

Only 36 individuals were screened in 2010 (Table 2). Consequently, it is not possible to assess whether the prevalence and intensity of infection changed from 2010 to 2021–2023. Additional File 1 Fig. S6 and Tables S4, S5, and S6 provide descriptive statistics.

**Table 2:** Skin microfilariae density across all and across mf-positive individuals without prior ivermectin treatment in Health Zones Logo and Nyarambe

Health Zone	All individuals					mf-positive individuals				
Period	*N*	Mean SmfD	SD SmfD	Min SmfD	Max SmfD	*N*	Mean SmfD	SD SmfD	Min SmfD	Max SmfD
Logo										
2010	1373	24.3	35.52	0	299.4	1049	31.8	37.59	0.1	299.4
Adults	1265	25.4	36.42	0	299.4	970	33.1	38.40	0.1	299.4
Adolescents	108	11.8	18.37	0	73.5	79	16.1	19.80	0.1	73.5
2021–2023	7547	1.1	6.44	0	177.8	625	13.1	18.54	0.1	177.8
Adults	5517	1.4	7.33	0	177.8	561	13.7	19.02	0.1	177.8
Adolescents	2030	0.3	2.62	0	66.6	64	7.9	12.62	0.1	66.6
Nyarambe										
2010 Adults	36	10.6	24.39	0	135.6	26	14.6	27.77	0.1	135.6
2021–2023	1047	0.4	2.89	0	53.8	30	13.2	11.21	0.2	53.8
Adults	690	0.6	3.54	0	53.8	30	13.2	11.21	0.2	53.8
Adolescents	357	0.0	0.00	0	0.0	0				

### Logo Health Zone

#### Skin microfilariae density across all health areas

**Fig. 4:**
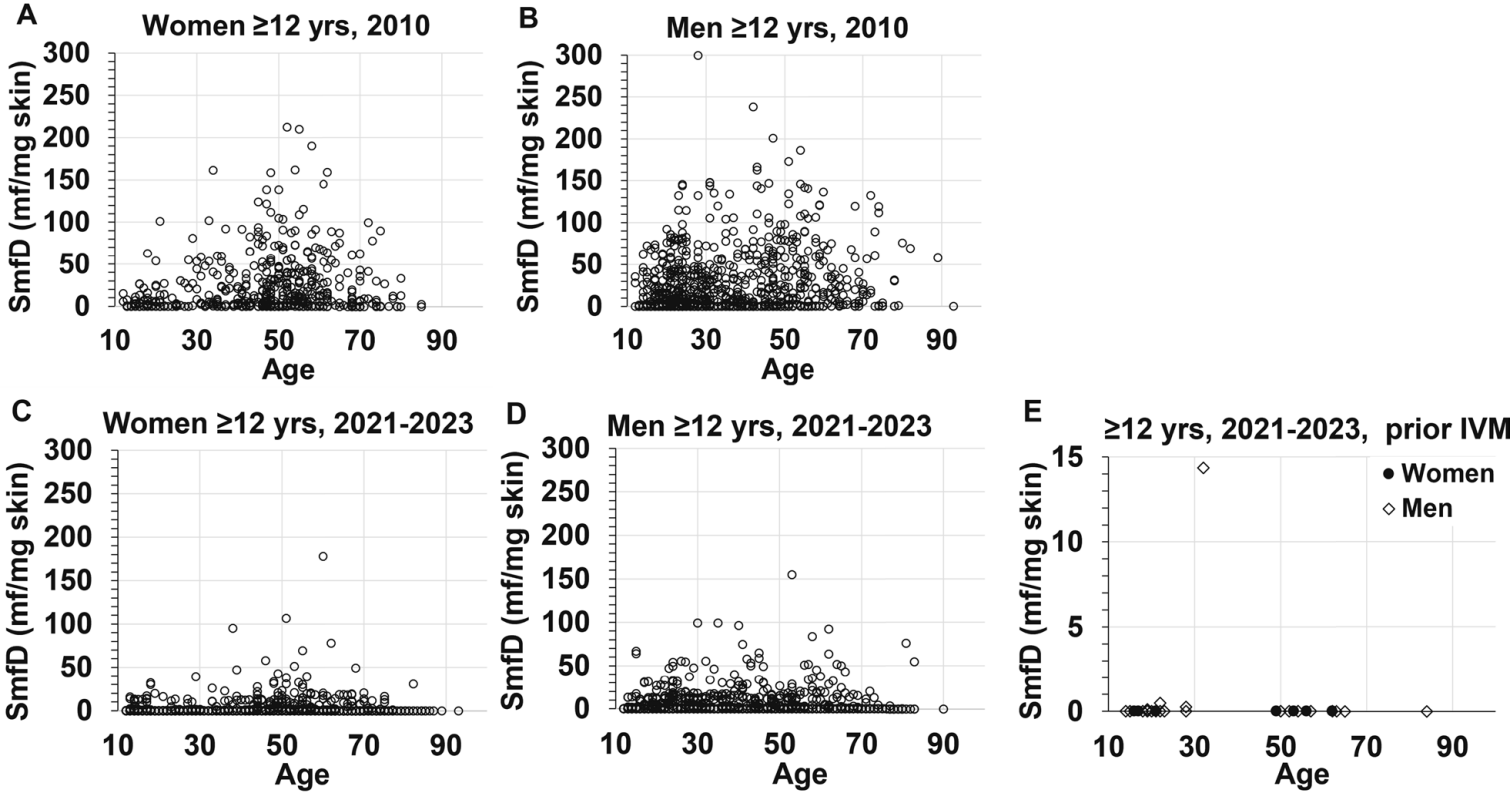
Skin microfilariae density among all women (**A**) and men (**B**) screened in 2010, and women (**C**) and men (**D**) screened in 2021–2023 who had no prior ivermectin treatment, and **E** women and men screened in 2021–2023 who reported ivermectin treatment between 2 and 1800 days before screening in Logo Health Zone. SmfD, skin microfilariae density; mf, microfilariae, **A** *n* = 506, **B** *n* = 867, **C** *n* = 3525, **D** *n* = 4022, **E** women *n* = 8, men *n* = 21

**Fig. 5:**
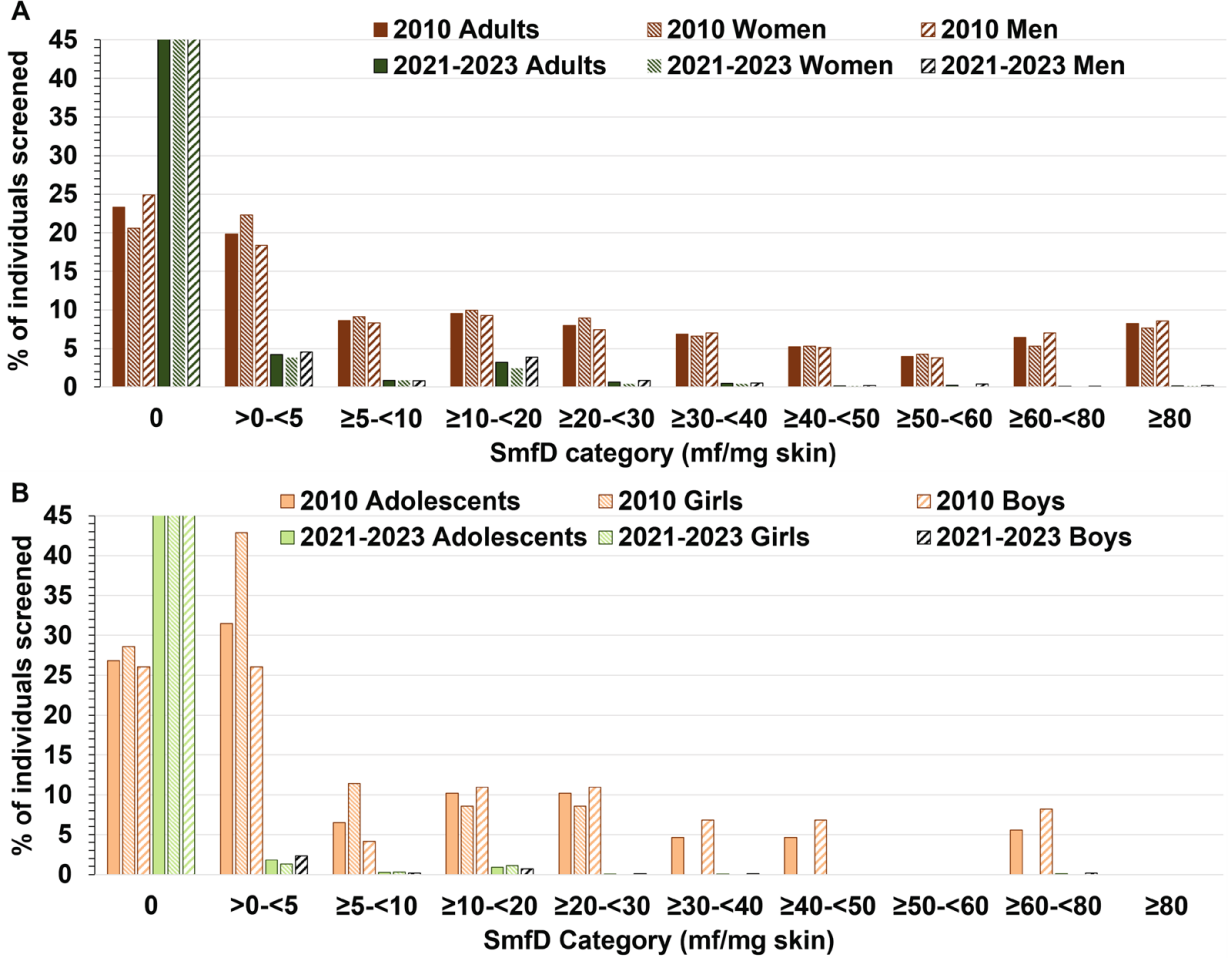
Distribution of skin microfilariae density in 2010 and 2021–2023 in the Health Zone Logo among adults and adolescents without prior ivermectin treatment. **A** Adults *N* = 1265 in 2010 and *N* = 5517 in 2021–2023, **B** Adolescents *N* = 108 in 2010 and *N* = 2030 in 2021–2023

Figure 4 shows the SmfD in each individual screened in 2010 and 2021–2023 by age and gender. Mean ± standard deviations SmfD across all individuals without ivermectin treatment history was 24 ± 35.5 in 2010 and 1 ± 6.4 in 2021–2023 (Table 2). Mean ± standard deviation SmfD across all mf-positive individuals without ivermectin treatment history was 32 ± 37.6 in 2010 and 13 ± 18.5 in 2021–2023 (Table 2). In 2010, no mf were detected in the skin snips of only 23.3% and 26.9% of adults and adolescents, while these percentages were 89.8% and 96.8% in 2021–2023, respectively (Fig. 5). Additional File 1 Tables S4, S5, and S6 provide further details.

The odds ratio (OR) for infection (mf positivity) was 3.101 (CI [2.053, 4.713], *P* < 0.001) and 0.024 (CI [0.019, 0.030], *P* < 0.001) in 2010 and 2021–2023, respectively. In 2021–2023, males were statistically significantly more likely to be infected than women (OR 1.643, CI [1.386, 1.952], *P* < 0.001) and the odds of being infected increased with age (OR 1.030, CI [1.025, 1.034], *P* < 0.001). Prior ivermectin treatment did not significantly impact the odds of being mf-positive (OR 1.348, CI [0.388, 3.602, *P* > 0.1). In contrast, in 2010, the ORs for males versus females and increase with age were not statistically significant (OR 0.836, CI [0.634, 1.099] *P* > 0.1 and OR 1.004, CI [0.996, 1.012] *P* > 0.1, respectively).

The *x*-axis maximum was set to 45% rather than 100% to display the highest applicable percentage (97.3%) since such scaling renders the difference in the percentages in the SmfD categories above 0 harder to see. The percentage of all adults, adult women, and adult men in 2021–2023 without detectable SmfD levels was 89.8%, 91.6%, and 88.4%, respectively. The percentage of all adolescents, girls, and boys in 2021–2023 without detectable SmfD levels was 96.8%, 97.3%, and 96.4%, respectively.

#### Prevalence and intensity of infection by Health Area of residence

Volunteers living in the Health Areas Draju and Kanga represented 84% (1149/1373) and 12% (161/1373) of those screened in 2010 and 53% (4015/7547) and 35% (2673/7547) of those screened without prior ivermectin treatment in 2021–2023, respectively. Across these adults and adolescents, infection prevalence dropped from 79% (908/1149) to 9% (363/4015) in Draju and from 69% (111/161) to 9% (231/2673) in Kanga and mean SmfD from 26.2 to 1.2 in Draju and from 17.1 to 1.1 in Kanga (Table 3).

**Table 3:** Skin microfilariae density across all and all mf-positive individuals without prior ivermectin treatment in Health Areas Draju and Kanga

Health Area	All					mf-positive				
Period	*N*	Mean SmfD	SD SmfD	Min SmfD	Max SmfD	*N*	Mean SmfD	SD SmfD	Min SmfD	Max SmfD
Draju										
2010	1149	26.2	36.71	0	299.4	908	33.1	38.41	0.1	299.4
Adults	1057	27.3	37.65	0	299.4	838	34.4	39.27	0.1	299.4
Adolescents	92	13.4	19.40	0	73.5	70	17.6	20.53	0.1	73.5
2021–2023	4015	1.2	6.70	0	177.8	362	13.0	18.61	0.1	177.8
Adults	2803	1.6	7.80	0	177.8	324	13.5	19.13	0.1	177.8
Adolescents	1212	0.3	2.64	0	66.6	38	8.2	12.70	0.1	66.6
Kanga										
2010	161	17.1	29.21	0	200.8	111	24.8	32.38	0.1	200.8
Adults	151	18.1	29.88	0	200.8	107	25.5	32.75	0.1	200.8
Adolescents	10	2.3	5.43	0	16.9	4	5.9	7.81	0.3	16.9
2021–2023	2673	1.1	6.20	0	99.2	231	13.2	16.90	0.1	99.2
Adults	1967	1.5	7.00	0	99.2	205	13.9	17.24	0.1	99.2
Adolescents	706	0.3	2.79	0	64.1	26	7.5	12.75	0.1	64.1

#### Prevalence and intensity of infection by village of residence

In 18 villages, screening occurred in both 2010 and 2021–2023. Participants from these villages represented 95% (1302/1373) and 76% (5738/7547) of participants without prior ivermectin treatment. Data from all 18 villages are provided in Additional File 1 Table S5.

**Fig. 6:**
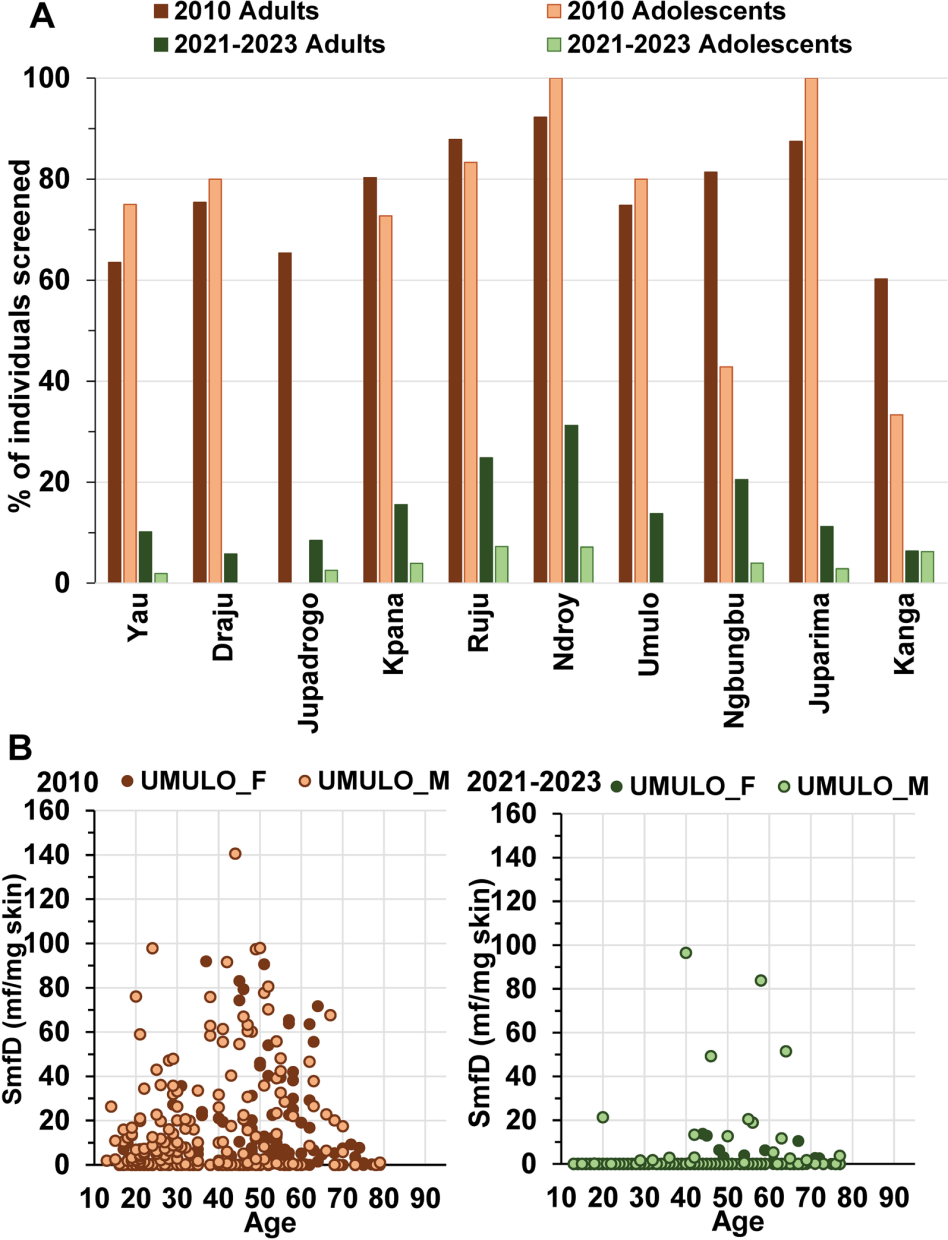
**A** Percentage of mf-positive adults and adolescents in 2010 and 2021–2023 in the 10 villages in Logo Health Zone where at least 20 individuals without prior ivermectin treatment were screened in both periods. **B** Skin microfilariae density in each individual without prior ivermectin treatment screened in 2010 and in 2021–2023 in Umulo by age and gender. F, women; M, men. **A** In 2010, the number of adults screened in 2010 was Yau 67, Draju 66, Jupadrogo 26, Kpana 97, Ruju 144, Ndroy 73, Umulo 324, Ngbungbu 136, Juparima 25, Kanga 97, and in 2021 2023 was Yau 673, Draju 474, Jupadrogo 193, Kpana 283, Ruju 218, Ndroy 106, Umulo 324, Ngbungbu 147, Juparima 682, Kanga 394. **B** *N* = 324 in both 2010 and 2021–2023

In 10 of these 18 villages, more than 20 volunteers were screened in 2010 and 2021–2023. Figure 6A illustrates the drop in prevalence of mf-positive individuals from 2010 to 2021–2023 in these villages. Figure 6B and Additional File 1 Fig. S7 show for Umulo and the other nine villages, respectively, that infection prevalence and intensity dropped across genders and age.

In these 10 villages, the odds ratio (OR) for infection (mf-positivity) was 3.274 (CI [2.111, 5.116], *P* < 0.001) and 0.019 (CI [0.014, 0.026], *P* < 0.001) in 2010 and 2021–2023, respectively. In 2021–2023, males were statistically significantly more likely to be infected than women (OR 1.678, CI [1.321, 2.138], *P* < 0.001) and the odds of being infected increased with age (OR 1.040, CI [1.034, 1.047], *P* < 0.001). In contrast, in 2010, the ORs for males versus females and increase with age were not statistically significant (OR 0.773, CI [0.574, 1.035], *P* = 0.1 and OR 1.005, CI [0.997, 1.013], *P* > 0.1, respectively). These ORs are similar to those obtained across all individuals screened in Logo Health Zone. The OR for being infected in these 10 villages in 2021–2023 compared with in 2010 was 0.003 (CI [0.001, 0.007], *P* < 0.001). In 2021–2023, males had a relatively higher risk of being infected than in 2010 (OR 1.966, CI [1.333, 2.911], *P* < 0.001) and the odds of being infected increased more with age than in 2010 (OR:1.036, CI [1.025, 1.047], *P* < 0.001).

#### Prevalence and intensity of infection by river basin

**Fig. 7:**
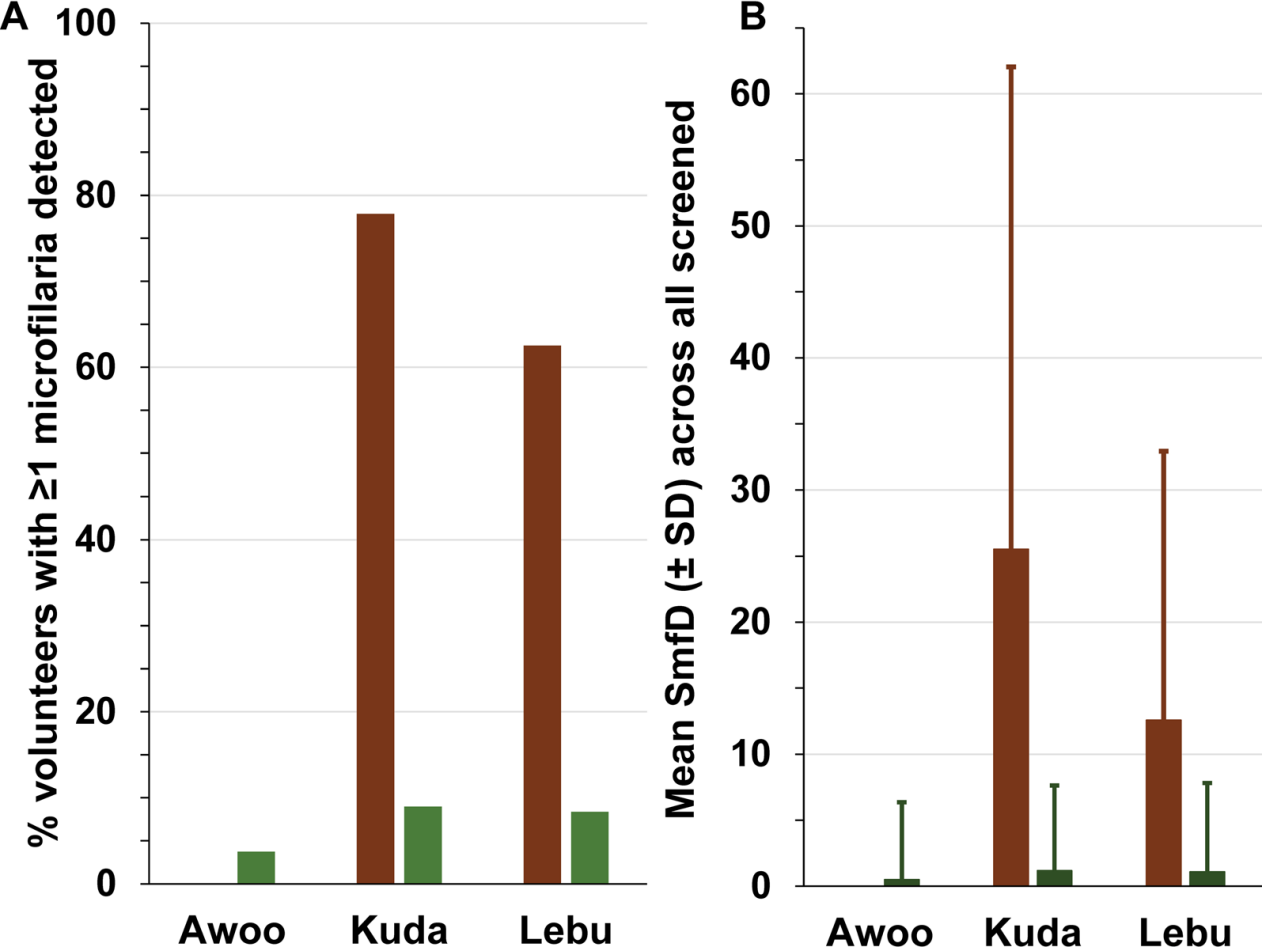
Prevalence and mean skin microfilariae density in 2010 and in 2021–2023 by river basin among individuals without prior ivermectin treatment living in the Logo Health Zone. **A** Percentage of volunteers who did not report prior ivermectin treatment screened with ≥ 1 microfilaria detected. **B** Arithmetic mean skin microfilariae density across all who did not report prior ivermectin treatment. Number screened in 2010 (brown) and 2021–2023 (green), respectively: Awo 0 and 858, Kuda 1245 and 5671, and Lebu 128 and 1017

Rivers and river basins are an epidemiologically more relevant unit for “river blindness” than administrative units such as villages, Health Areas and Zones, or countries (see, e.g., the 2024 WHO handbook for onchocerciasis elimination mapping [64]. Figure 7 shows the drop in infection prevalence and intensity from 2010 to 2021–2023 across all individuals screened living in the Kuda and Lebu river basin. No conclusions are possible for the Awo basin because no individual screened in 2010 lived in a village in the Health Zone Logo in that basin.

## Discussion

The SmfD of individuals living in the Health Zone Logo, whether analyzed overall, by age group, or by Health Area, village, or river basin when numbers screened allowed, showed a substantial drop in the prevalence and intensity of infection from 2010 to 2021–2023. No conclusions can be drawn for the Health Zone Nyarambe because only 36 individuals were screened in 2010. Given that people living in the Health Zone Logo can move freely into the Health Zone Nyarambe and might actually move regularly into that Health Zone for field work close to breeding sites, it would be surprising if prevalence and intensity of infection had not changed in the Health Zone Nyarambe independent of the ivermectin treatments for LF control in that Health Zone.

Neither dataset was obtained to sample age- and gender-representative sections of the population to assess infection levels (Table 1, Additional File 1 Fig. S5). The sample size and area covered are, however, large and the method used is precise compared with other evaluations (Additional File 1 Table S7). Our analysis could not account for (1) a “participant self-selection bias” due to potential participants being told that people with “too few worms in their skin” cannot participate in the study in 2010 (the P3 study inclusion criteria required ≥ 10 SmfD [29, 37, 38]) but not in 2021–2023 (Fig. 2), which might have discouraged those considering themselves uninfected from participating in screening in 2010, and (2) that where people live is not where they work the fields and might get infected. These limitations are, however, unlikely to explain the decrease in infection prevalence and intensity the SmfD data show.

Given that ivermectin was not distributed in the Health Zone Logo between 2010 and 2021–2023, ivermectin distribution cannot explain this decrease (nor can the single dose of moxidectin or ivermectin that 472 infected individuals across 17 villages received in the P3 study in 2010 to January 2011). The 2010 data show that the Health Zone Logo met the criteria for hyperendemicity [11, 65, 66]. Considering the data and modeling showing that long-term CDTI with high treatment coverage in hyperendemic areas themselves is needed to significantly reduce infection prevalence and intensity [25, 59, 67], ivermectin distribution in the Health Zone Nyarambe in 2016–2019 and 2021–2022 is also very unlikely to have driven the decrease in infection prevalence and intensity in the Health Zone Logo. Consequently, the most likely cause is decreasing infective vector biting rates. This leads to decreasing numbers of young macrofilariae, i.e., a shift in the macrofilariae population toward older macrofilariae approaching or beyond the end of their reproductive lifespan and thus decreasing numbers of mf-producing macrofilariae. The OCP showed these effects when comparing the macrofilariae from volunteers in villages after 7–10 years of vector control and villages without vector control. This research also found lower reproductive capacity in older compared with younger macrofilariae [68], which further contributes to lowering host SmfD [57, 59]. While following up MDGH-MOX-3001 and MDGH-MOX-3002 participants in seven villages (Kpana, Ndroy, Rudju, Mbesi, Yau, Ngungbu, and Jabi) whose inhabitants work the fields in the Kuda river basin, village leaders told FNN and the nurses: (1) that blackfly nuisance had declined and blackfly bites decreased from several/day to several/week over the past approximately 10 years, (2) blackfly nuisance, previously present throughout the year, is now restricted to the two rainy seasons. The authorization provided by one of the “grands chefs” to clear forests in the Kuda basin valleys for agricultural use and to reduce blackfly nuisance has left little shade cover for blackflies (Additional File 1 Fig. S3). We are not aware of any larviciding or other vector control interventions by national or provincial authorities.

In their report of entomological evaluations in the area, Post et al. [46] point out that: (1) *S. neavai* may have been the main or only anthropophilic vector in the past and disappeared following land use changes that reduced shade cover that species needs; (2) the main species identified between 2015 and 2018 was *S. dentulosum*, a species with unarmed cibarium, (3) *S. dentulosum* does not regularly feed on humans and had never been implicated in *O. volvulus* transmission; (4) given that 30% and 11% of 155 *S. dentulosum* caught via human landing catches were *O. volvulus* infected and infective, respectively, *S. dentulosum* is a probable vector. Its efficiency as a vector is unknown; (5) further investigation is needed into the role of *S. vorax* in *O. volvulus* transmission given that only four specimens were available to assess *O. volvulus* infection and infectivity [46].

Publicly available results of other evaluations in and around our study area (Additional File 1 Table S7) include those of the 2002 REA (Additional File 1 Table S1, Figs. S2 and S4), the 2015 DRC MoH parasitological or serological evaluations, and research studies conducted from 2016 to 2018 on onchocerciasis-associated epilepsy [69–71]. Comparison of these data with ours cannot provide further insight into trends in parasite transmission for methodological reasons. The correlation between REA-based nodule prevalence and prevalence of mf-positive individuals in the general population is not very tight, and the skin mf prevalence data used to establish this correlation were obtained with two snips [66]. Two snips, typically used in prevalence surveys and studies not aimed at assessing the efficacy of anti-onchocercal drugs (including in the studies on onchocerciasis-associated epilepsy [69–72]), provide less sensitivity for detecting mf-positive individuals than four snips [63, 73]. Two of the studies by Mandro et al. [70, 71] included only persons with epilepsy. The long-postulated association between *O. volvulus* infection and risk of epilepsy has been confirmed [74–85]. Consequently, infection prevalence in persons with epilepsy cannot be compared with our data. The third study by Mandro et al. [72] included 65 persons without epilepsy from the Village Draju. The age data that Mandro et al. provided suggest that an estimated 20–45% of these 65 individuals were younger than our study population. Given that the prevalence of *O. volvulus* infection increases with age [86–88], this compromises comparison of their and our data. Nevertheless, Additional File 1 Table S8 includes the prevalence of infection calculated only on the basis of our data from the iliac crests. Assays for the presence of immunoglobulin G4 against the *O. volvulus* antigen Ov16 (Ov16 IgG4) detect both current and past infections. The time to seroconversion in both directions is unknown, and not everybody infected might seroconvert. A WHO advisory committee review of large-scale evaluations of Ov16 IgG4 prevalence identified discrepancies between results with different assays and concerns about false-positive and false-negative results. Thus, both methodological and biological factors can contribute to discordance between mf-positivity and Ov16 IgG4 positivity in the same individual and differences in infection prevalence estimated parasitologically or serologically [89–98].

When considering what the reduction in infection prevalence and intensity that we identified indicates about current transmission levels and prospects for elimination of transmission without interventions, it needs to be taken into account that the SmfDs measured reflect past transmissions. These transmissions occurred approximately 2–15 years earlier because (1) the pre-patent period is estimated at 12–18 months [58, 59, 99, 100] and it may take up to 3 years from an infective bite to the resulting macrofilaria(e) producing detectable mf levels [58, 59, 101], (2) the estimated reproductive life span of the macrofilariae is 10 ± 3 years [56–59], and (3) the estimated microfilariae life span is around 12–15 months [58, 59, 102]. To assess current transmission, extensive collection of anthropophilic vectors and determination of their infectivity is required.

While our data provide no information on current transmission levels, they show that transmission decreased significantly resulting in the macrofilariae age distribution among those screened in 2021–2023 versus in 2010, being shifted toward older and less reproductively capable macrofilariae. It is unknown whether older macrofilariae approaching the end of their reproductive lifespan have a different susceptibility to anti-onchocercal drugs (whether ivermectin, moxidectin, or drugs currently in development [39]) than younger macrofilariae. The transmission models ONCHOSIM and EpiOncho, used to estimate time to elimination with different strategies, consider macrofilaria age-dependent reproductive capacity (additional files to [59]) but to our knowledge not age-dependent drug susceptibility. Comparative analysis of the 12- and 18-month efficacy data from the P3 study in this area [29] and study MDGH-MOX-3001 may provide an opportunity to evaluate age dependency of macrofilariae susceptibility to ivermectin and moxidectin. This may be a unique opportunity given that evaluations in areas under CDTI [103] do not allow to differentiate the CDTI-driven reduction in transmission and macrofilariae age distribution and the cumulative effect of ivermectin treatment on macrofilariae reproductive capacity [59]. Estimates of macrofilariae-age-dependent drug susceptibility could improve model predictions.

## Conclusions

In the Health Zone Logo, *O. volvulus* infection prevalence and intensity dropped substantially over around 11–13 years without health system-directed interventions. Given that SmfD, nodule prevalence, and Ov16 IgG4 reflect past transmissions, large-scale entomological evaluations are needed to assess current transmission levels and thus the extent to which the change in the vector species proposed by Post and coworkers [46] may result in elimination or in keeping transmission at very low levels. Given that reduction in transmission, whether due to “natural causes” (e.g., land use or climate change), vector control, or large-scale treatment with current anti-onchocercal drugs, shifts macrofilariae age distribution, comparing ivermectin and moxidectin efficacy in the ongoing and the P3 study might help determine whether macrofilariae drug susceptibility changes with age. If it does, inclusion of relevant parameters in the transmission models could improve the accuracy of the modeled time to elimination.

## Supplementary Information

Supplementary Material 1. Table S1 GPS coordinates for villages in which individuals were screened in 2010 and/or 2021-2023. Fig. S1 Aires de Santé where participants lived with dwelling and major roadways in 2021-2023. Fig. S2 Overview of study area. Fig. S3 Photos of study area taken in 2022–2024 by T.O. Ukety and F.N. Nyisis. Table S2 Results of the rapid epidemiological assessment conducted in 2002 as per the site-level ESPEN database (accessed on 14 September 2024) with current Zone and Aire de Santé and APOC CDTI Project. Fig. S4 Map of 2002 rapid epidemiological assessment results. Table S3 CRMT capacities, research experience, and diseases in the area. Fig. S5 Percentage and number of individuals screened (lower panel) by age screened in 2010 and 2021–2023 in ZdS Logo and in 2021–2023 in the ZdS Nyarambe. Fig. S6 Skin microfilariae density among all women and men (A) screened in 2010, and women (B) and men (C) screened in 2021–2023 who had no prior ivermectin treatment and (D) women and men reporting ivermectin treatment between 2 and 1800 days before screening in ZdS Nyarambe. Table S4 Number of participants without prior IVM treatment screened in 2010 and 2021-2023 by age group, gender, and skin microfilariae density category by Zone de Santé. Table S5 Number of adults and adolescents without prior ivermectin treatment screened in 2010 and in 2021–2023 with and without detectable SmfD. Fig. S7 Skin microfilariae density among volunteers in the 10 villages where at least 20 individuals without prior ivermectin treatment were screened in 2010 and in 2021–2023 by age and gender. Table S6 Descriptive statistics of SmfD for mf-positive individuals without prior ivermectin treatment screened in 2010 and in 2021–2023. Table S7 Publicly available data on onchocerciasis prevalence in and around the Zone de Santés Nyarambe and Logo. Table S8 Prevalence of *O. volvulus* infection by village based on skin mf densities from only the two iliac crests. References. Abbreviations.

## Data Availability

Data supporting the main conclusions of this study are included in the manuscript.

## Abbreviations

APOC: African Programme for Onchocerciasis Control
CDTI: Community-directed treatment with ivermectin
CI: 95% confidence interval
CRMT: Centre de Recherche en Maladies Tropicales de l’Ituri (in Rethy, Ituri)
DRC: Democratic Republic of the Congo
G-FDA: Food and Drugs Authority of Ghana
MDAi: Mass drug administration of ivermectin
MDGH: Medicines Development for Global Health
mf: Microfilaria
MoH: Ministère de la Santé Publique, Hygiène et Prévention of the Democratic Republic of the Congo
OR: Odds ratio
P3: Phase 3
REA: Rapid epidemiological assessment
SmfD: Skin microfilariae density (mf/mg skin)
SSA: Sub-Saharan Africa
US-FDA: Food and Drug Administration of the United States of America (USA)
WHO: World Health Organization
WHO/ESPEN: WHO Expanded Special Project for Elimination of Neglected Tropical Diseases
WHO/TDR: UNICEF/UNDP/World Bank/WHO Special Programme for Research and Training in Tropical Diseases
