# Supplementary material for: Onchocerca volvulus infection prevalence and intensity in Logo and Nyarambe Health Zones in Ituri, Democratic Republic of the Congo in 2010 and in 2021–2023: results of screening for clinical trials of moxidectin versus ivermectin
Source: Parasit Vectors. 2026 Mar 16;19:140. doi: 10.1186/s13071-025-07199-8 (PMC13037001; doi:10.1186/s13071-025-07199-8)
Supplement: Supplementary file 1 — Supplementary Material 1. Table S1 GPS coordinates for villages in which individuals were screened in 2010 and/or 2021-2023. Fig. S1 Aires de Santé where participants lived with dwelling and major roadways in 2021-2023. Fig. S2 Overview of study area. Fig. S3 Photos of study area taken in 2022–2024 by T.O. Ukety and F.N. Nyisis. Table S2 Results of the rapid epidemiological assessment conducted in 2002 as per the site-level ESPEN database (accessed on 14 September 2024) with current Zone and Aire de Santé and APOC CDTI Project. Fig. S4 Map of 2002 rapid epidemiological assessment results. Table S3 CRMT capacities, research experience, and diseases in the area. Fig. S5 Percentage and number of individuals screened (lower panel) by age screened in 2010 and 2021–2023 in ZdS Logo and in 2021–2023 in the ZdS Nyarambe. Fig. S6 Skin microfilariae density among all women and men (A) screened in 2010, and women (B) and men (C) screened in 2021–2023 who had no prior ivermectin treatment and (D) women and men reporting ivermectin treatment between 2 and 1800 days before screening in ZdS Nyarambe. Table S4 Number of participants without prior IVM treatment screened in 2010 and 2021-2023 by age group, gender, and skin microfilariae density category by Zone de Santé. Table S5 Number of adults and adolescents without prior ivermectin treatment screened in 2010 and in 2021–2023 with and without detectable SmfD. Fig. S7 Skin microfilariae density among volunteers in the 10 villages where at least 20 individuals without prior ivermectin treatment were screened in 2010 and in 2021–2023 by age and gender. Table S6 Descriptive statistics of SmfD for mf-positive individuals without prior ivermectin treatment screened in 2010 and in 2021–2023. Table S7 Publicly available data on onchocerciasis prevalence in and around the Zone de Santés Nyarambe and Logo. Table S8 Prevalence of O. volvulus infection by village based on skin mf densities from only the two iliac crests. References. Abbrevia [file 13071_2025_7199_MOESM1_ESM.pdf]

Additional File 1

Onchocerca volvulus infection prevalence and intensity in Logo and Nyarambe Health Zones in Ituri, Democratic Republic of the Congo in 2010 and in 2021-2023: Results of screening for clinical trials of moxidectin vs. ivermectin

Françoise N. Ngave[1*], Deogratias U. Wonyarossi[1*], Germain M. Abhafule[1], Joël L. Mande[1], Amos Nyathirombo[1], Claude B. Uvon[1], Anuarite A. Raciu[1], Michel Mandro[1,2], Pascal T. Adroba[1,2], Tony O. Ukety[1], Innocent A. Mananu[1], Gisèle L. Abeditho[1], Jules U. Upenjirwoth[1], Carine M. Aliang'o[1], Jean de Dieu N. Unega[1], Maurice M. Nigo[1], Didier Bakajika[1], Jean-Paul U. Uvoyo[1], Germain L. Mambandu[1], Christine M. Halleux[3], Michel Vaillant[4], Anna Schritz[4], Beatriz Mosqueira[5], Mupenzi Mumbere[5], Sally Kinrade[5], Annette C. Kuesel[3**]

[1]Centre de Recherche en Maladies Tropicales, Rethy, DRC; [2] Division Provinciale de la Santé de l'Ituri (Ituri Provincial Health Division), Bunia, DRC; [3]World Health Organization Special Programme for Research and Training in Tropical Diseases (TDR), Geneva, Switzerland; [4] Competence Center for Methodology and Statistics, Luxembourg Institute of Health, Strassen, Grand Duchy of Luxembourg, [5]Medicines Development for Global Health, Melbourne, Australia.

* Joint first authors

**Corresponding author: kuesela@who.int, annette.kuesel@mailbox.org ORCID ID: 0000-0002-1696-1784

Table of Content

Table S1	GPS coordinates for villages in which individuals were screened in 2010 and/or 2021-2023	2
Fig S1	Aires de Santé (health area) where participants lived with dwelling and major roadways in 2021-2023	4
Fig S2	Overview of study area	5
Fig S3	Photos of study area taken in 2022-2024 by T.O. Ukety and F.N. Ngave	10
Table S2	Results of the Rapid Epidemiological Assessment conducted in 2002 as per the site-level ESPEN database (accessed on 14 September 2024) with current Zone and Aire de Santé and APOC CDTI Project	11
Fig S4	Map of 2002 rapid epidemiological assessment results	12
Table S3	CRMT capacities, research experience and diseases in the area	12
Fig S5	Percentage and number of individuals screened (lower panel) by age screened in 2010 and 2021-2023 in Zone de Santé (Health Zone) Logo and in 2021-2023 in the Zone de Santé Nyarambe	15
Fig S6	Skin microfilariae density among all women and men (A) screened in 2010, and women (B) and men (C) screened in 2021-2023 who had no prior ivermectin treatment and (D) women and men reporting ivermectin treatment between 2 and 1800 days before screening in the Zone de Santé (Health Zone) Nyarambe	16
Table S4	Number of participants without prior IVM treatment screened in 2010 and 2021-2023 by age group, gender and skin microfilariae density category by Zone de Santé (Health Zone)	17
Table S5	Number of adults and adolescents without prior ivermectin treatment screened in 2010 and in 2021-2023 with and without detectable SmfD	18

Fig S7 Skin microfilariae density among volunteers in the 10 villages where at least 20 individuals without prior ivermectin treatment were screened in 2010 and in 2021-2023 by age and gender 22
Table S6 Descriptive statistics of SmfD for mf positive individuals without prior ivermectin treatment screened in 2010 and in 2021-2023 23
Table S7 Publicly available data on onchocerciasis prevalence in and around the Zone de Santés Nyarambe and Logo 25
Table S8 Prevalence of individuals with >0 *O. volvulus* microfilariae detected in the two iliac crests by village 26
References 29
Abbreviations 30

Table S1 GPS coordinates for villages in which individuals were screened in 2010 and/or 2021-2023

Village	Latitude	Longitude	Screening in ≥ 12-year-old residents in
Awora	2.149422	31.00475	2010
Awura	2.170336	30.94661	2010
Bugo	2.053667	30.95173	2010
Buu Mission	2.159833	30.96908	2010
Cucu	2.07205	30.93612	2010
Jupahoy	2.145036	30.94722	2010
Jupalebe	2.159703	30.94873	2010
Jupaliri Gulukpa	2.147808	30.95815	2010
Jupanjaya	2.033444	30.95956	2010
Jupukelo	2.101611	30.95303	2010
Madi	2.161111	30.96919	2010
Mbraze	2.155556	31.00328	2010
Pacung	2.142547	30.99176	2010
Pamundu	2.159914	30.95423	2010
Raa	2.078139	30.92981	2010
Ulyeko	2.135578	30.95736	2010
Umulo1	2.134792	30.98717	2010
Draju	2.117621	30.96566	2010 and 2021-2023
Dyambu	2.061655	30.96504	2010 and 2021-2023
Jabi	2.089675	30.9868	2010 and 2021-2023
Jupadrogo	2.140581	30.96788	2010 and 2021-2023
Juparima	2.080844	30.95728	2010 and 2021-2023
Jupudero 2	2.146113	31.00382	2010 and 2021-2023
Kanga	2.074232	30.95317	2010 and 2021-2023
Kondu	2.120349	30.96664	2010 and 2021-2023
Kpana	2.108079	30.96659	2010 and 2021-2023
Kpanyi	2.135427	31.0016	2010 and 2021-2023
Loo	2.089522	30.96978	2010 and 2021-2023
Makala	2.113724	30.9627	2010 and 2021-2023
Mbesi	2.100096	30.9797	2010 and 2021-2023
Moo	2.064811	30.9759	2010 and 2021-2023
Ndroy	2.105226	30.97423	2010 and 2021-2023

Village	Latitude	Longitude	Screening in ≥ 12-year-old residents in
Ngbungbu	2.120581	30.97916	2010 and 2021-2023
Nzuru	2.12713	30.97725	2010 and 2021-2023
Ruju	2.110837	30.97555	2010 and 2021-2023
Umulo	2.136324	30.97671	2010 and 2021-2023
Yau	2.127804	30.96659	2010 and 2021-2023
Ang'al Unen	2.172634	31.00651	2021-2023
Drayi	2.177839	30.99493	2021-2023
Gbii	2.17642	31.01938	2021-2023
Jupafoyo	2.111444	31.00594	2021-2023
Jupajalbonyo	2.118448	31.00965	2021-2023
Jupajaza	2.1727	30.9945	2021-2023
Jupanyamoro	2.12845	31.00974	2021-2023
Jupasugu	2.1308	31.01002	2021-2023
Jupawalu	2.123071	31.01198	2021-2023
Jupawegi	2.119218	30.99672	2021-2023
Jupudero 1	2.117596	31.00642	2021-2023
Jupujanga	2.114981	31.00623	2021-2023
Jupuvuga	2.114032	31.00641	2021-2023
Jupuyuru	2.119781	31.00694	2021-2023
Madi Kaka	2.170743	30.98268	2021-2023
Nyodu	2.093644	30.96067	2021-2023
Paley	2.184625	31.00538	2021-2023
Thecer	2.167889	30.9759	2021-2023
Thedeja1	2.164615	30.99248	2021-2023
Thedeja2	2.177268	30.99661	2021-2023
Ucudo	2.177568	30.99661	2021-2023
Uryang'	2.16866	31.02094	2021-2023
Vuna	2.181087	31.02118	2021-2023
Wiloo	2.160501	30.99029	2021-2023
Wiraa	2.176012	30.9996	2021-2023

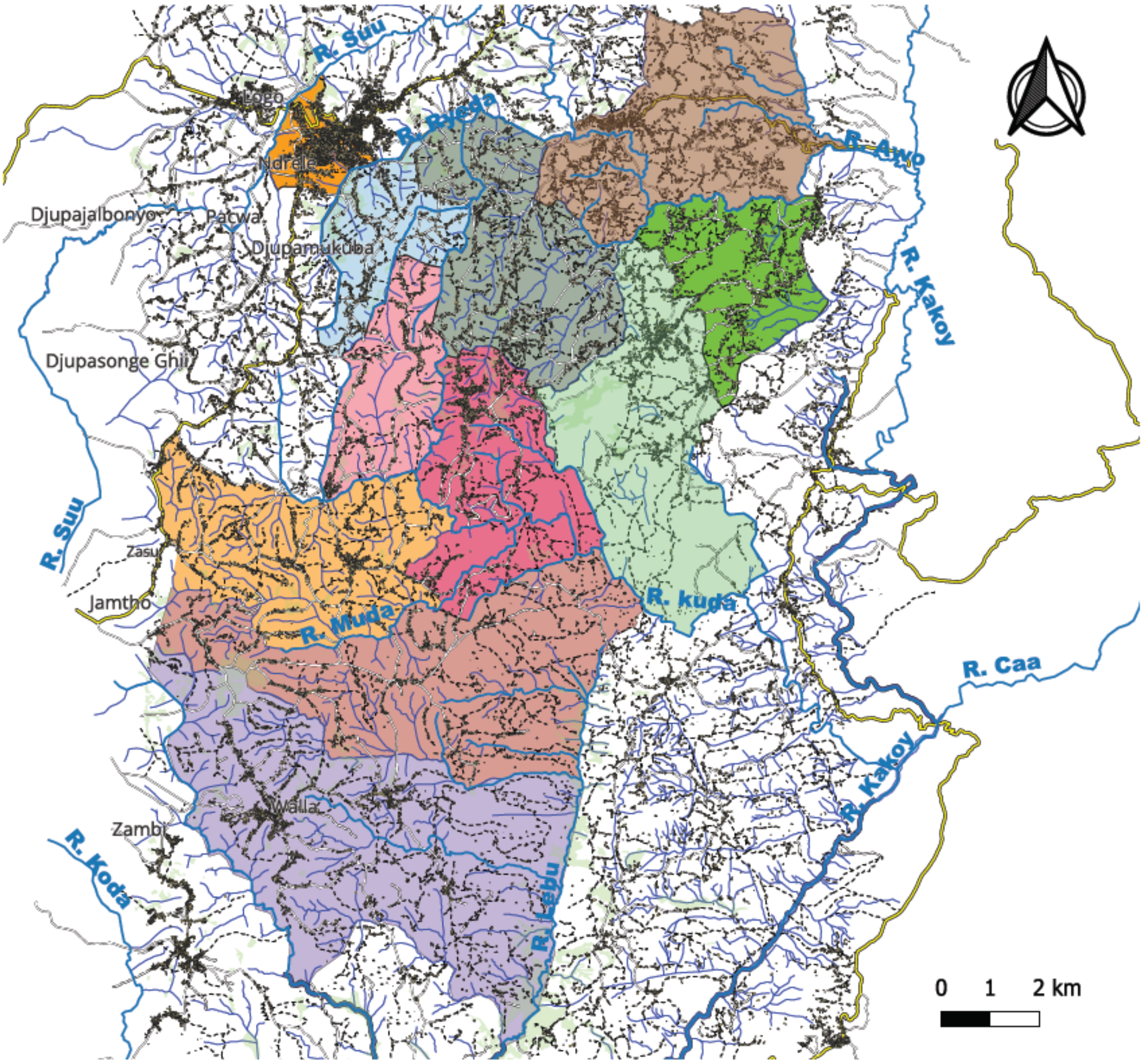

Fig S1 **Aires de Santé (health area) where participants lived with dwelling and major roadways in 2021-2023**

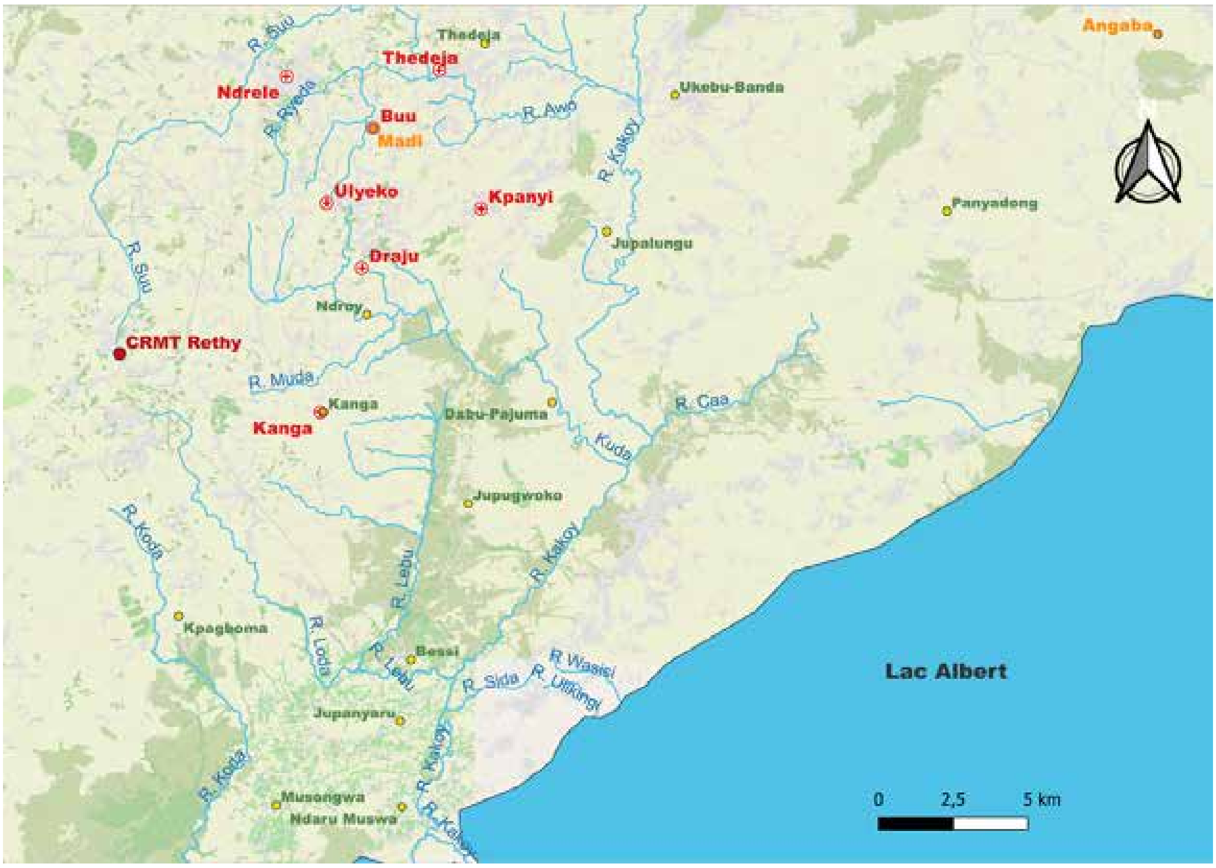

Fig S2 **Overview of study area**

Red crosses: Health centers (including the Health Center in Ndrele where the CRMT satellite office is located; red bullet: CRMT Rethy; Villages named in green: included in 2002 REA, villages named in orange: included in 2015 parasitological and serological evaluations. Background: Open Street View (date unknown)

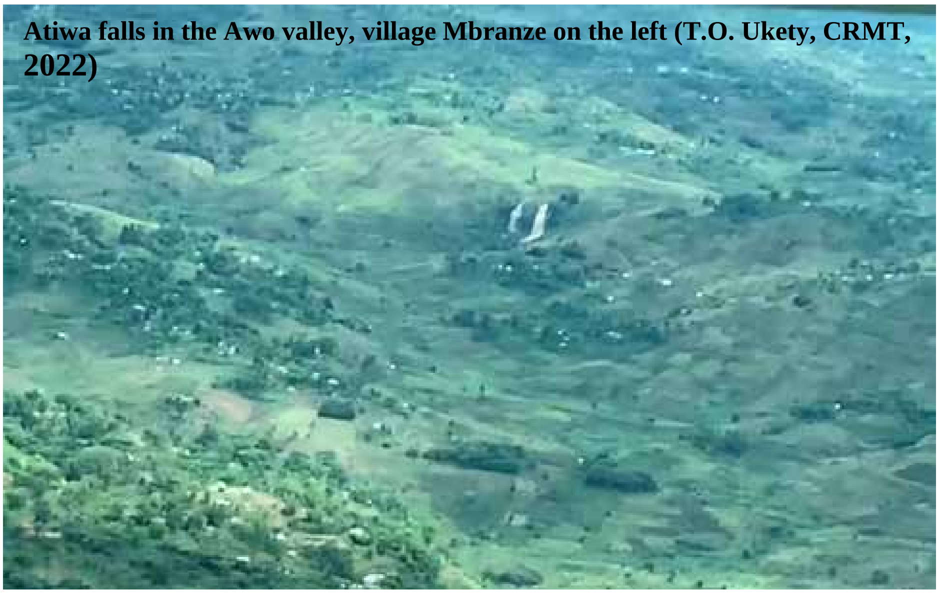

Atiwa falls in the Awo valley, village Mbranze on the left (T.O. Ukety, CRMT, 2022)

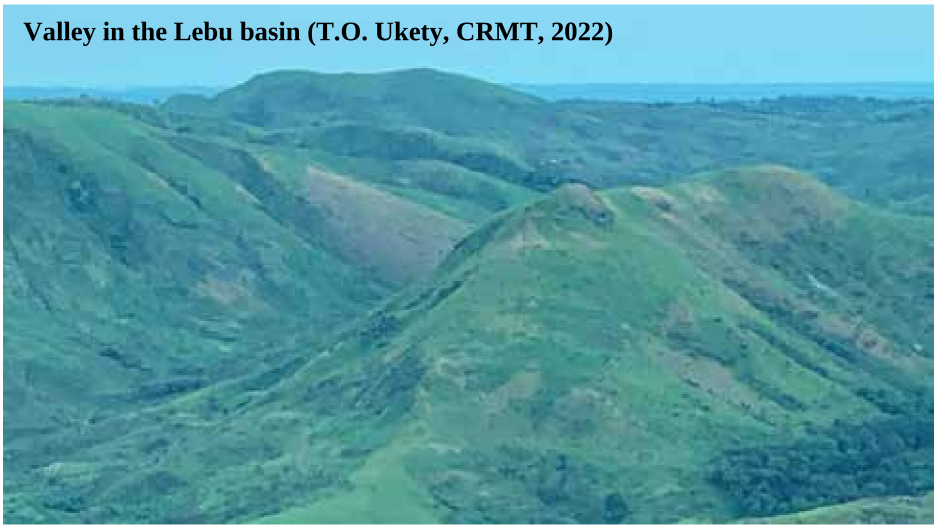

Valley in the Lebu basin (T.O. Ukety, CRMT, 2022)

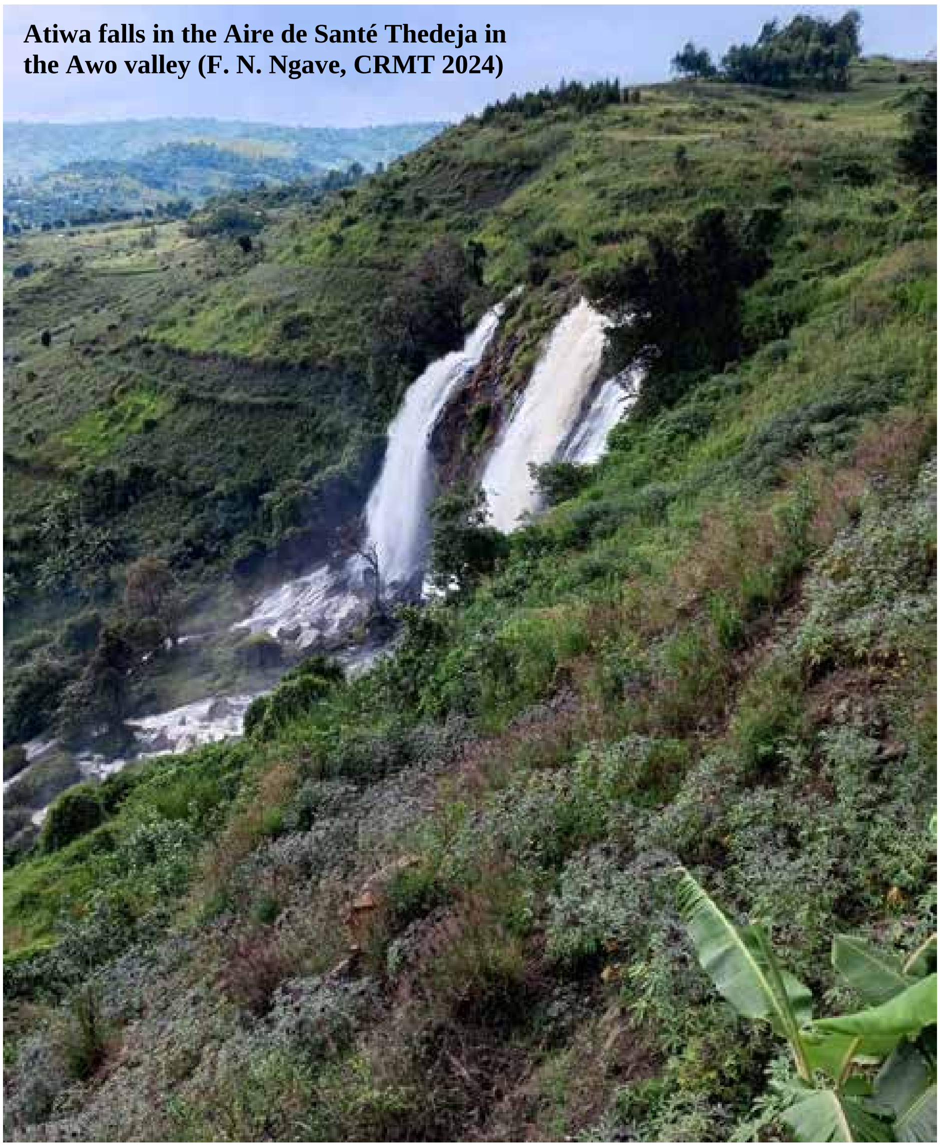

Atiwa falls in the Aire de Santé Thedeja in the Awo valley (F. N. Ngave, CRMT 2024)

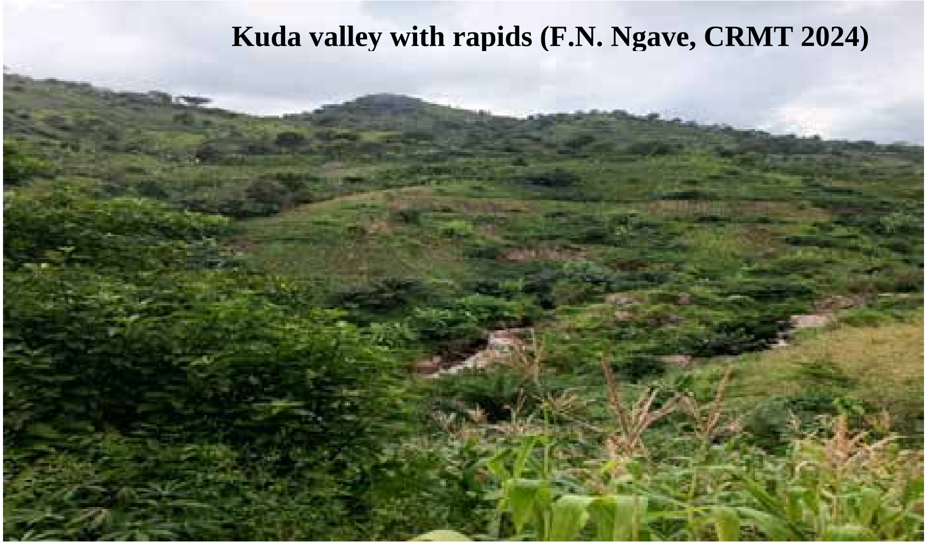

Kuda valley with rapids (F.N. Ngave, CRMT 2024)

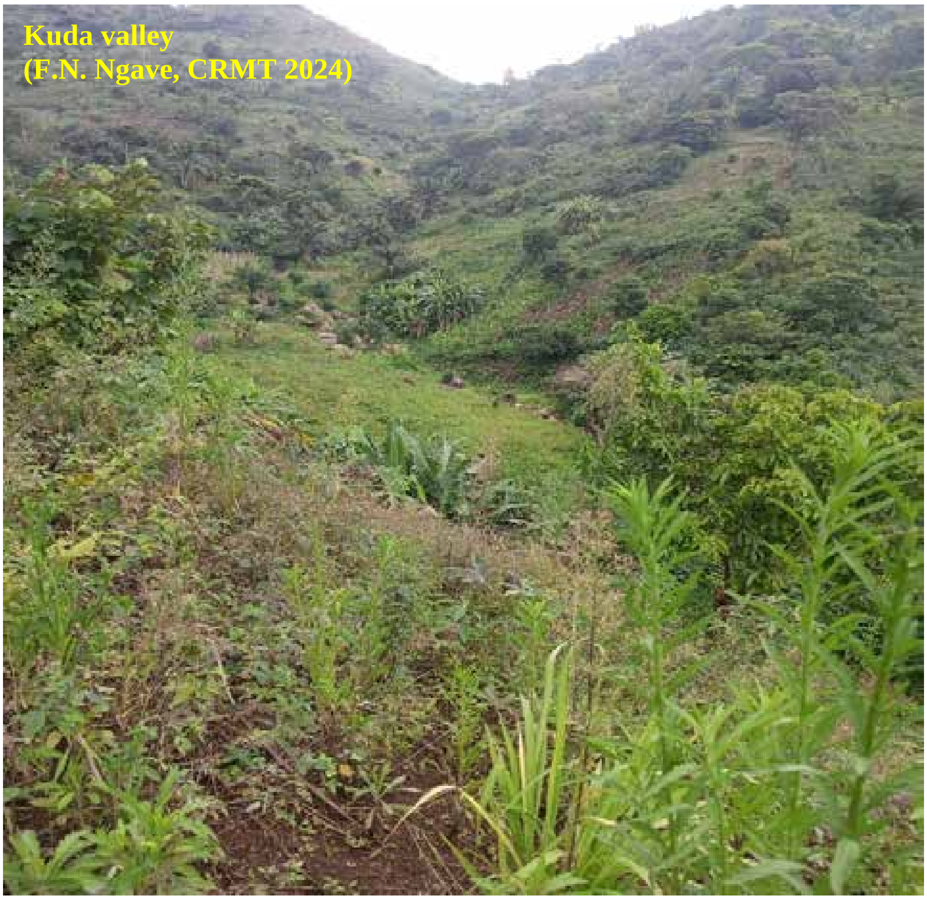

Kuda valley (F.N. Ngave, CRMT 2024)

Banana, corn, soja, palm tree fields in Kuda valley (F.N. Ngave, CRMT 2024)

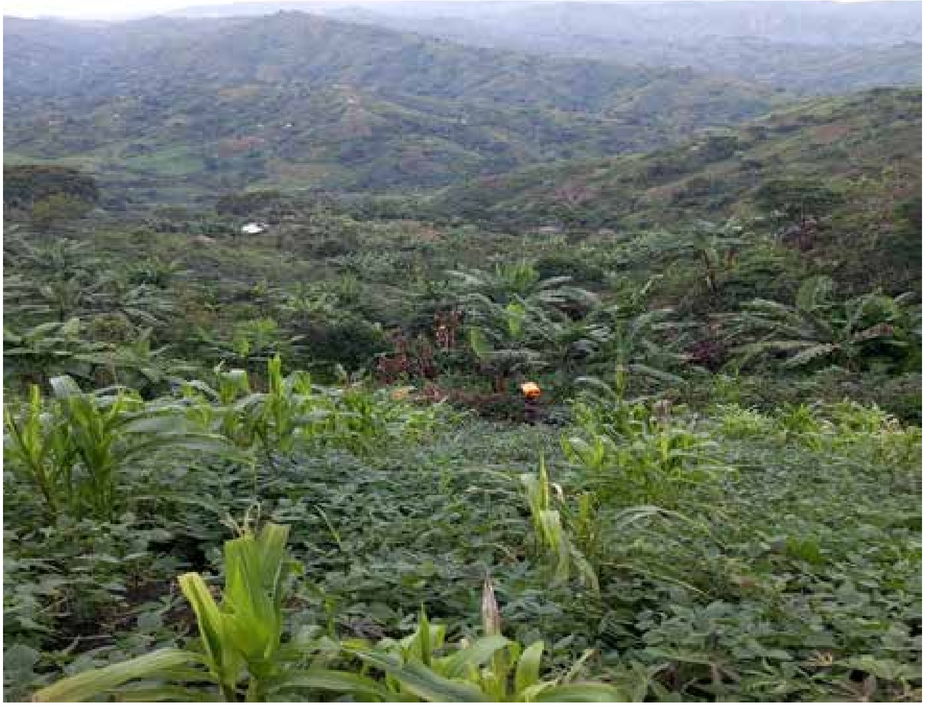

Palm tree, maniok, banana plants with hut used during field work in the Kuda valley (F.N. Ngave, CRMT 2024)

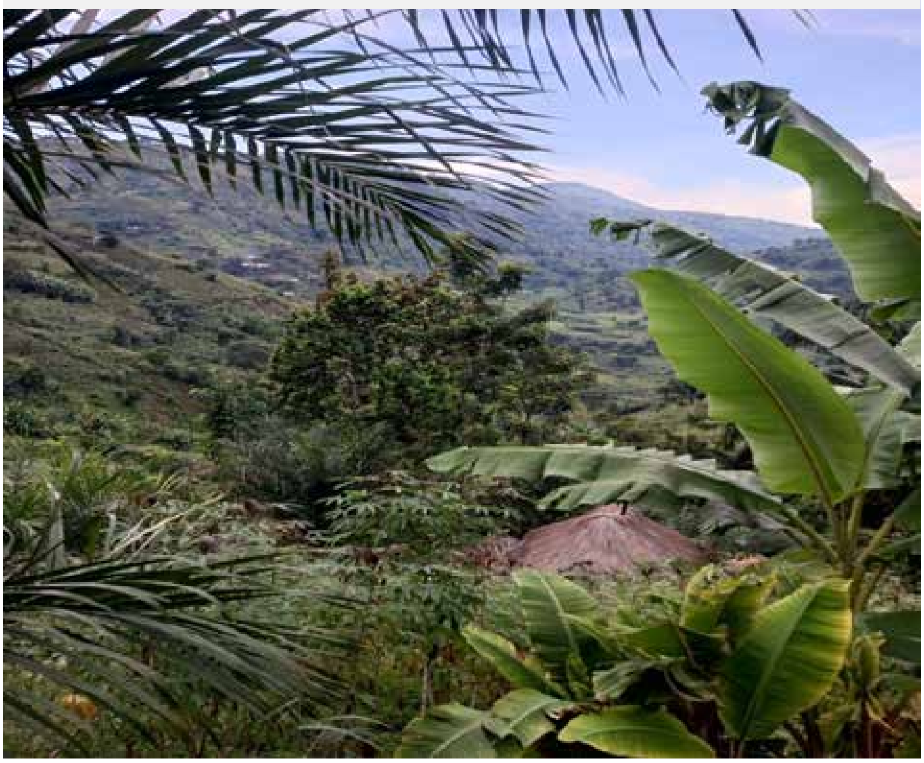

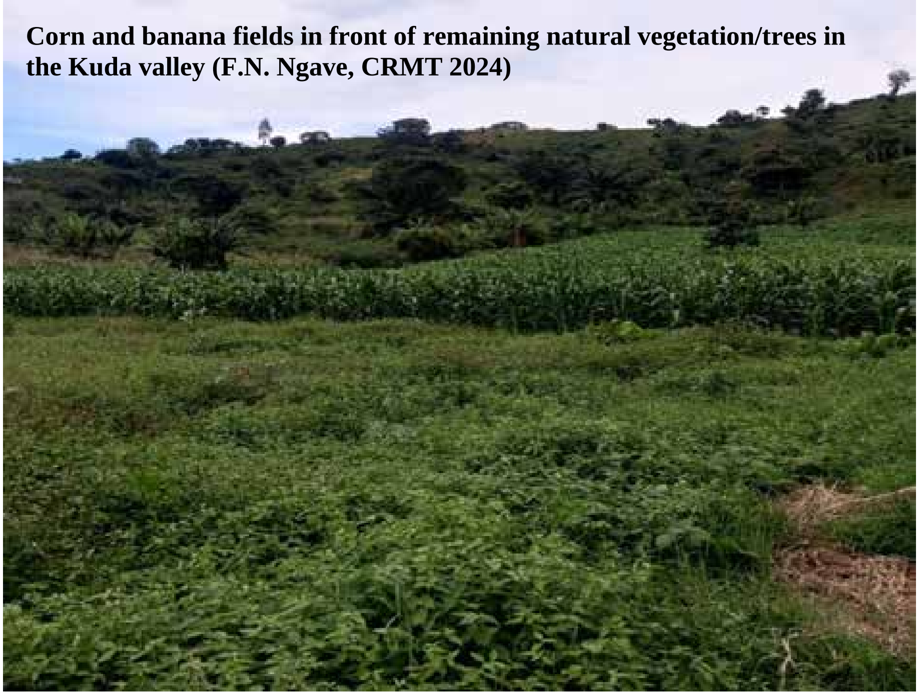

Corn and banana fields in front of remaining natural vegetation/trees in the Kuda valley (F.N. Ngave, CRMT 2024)

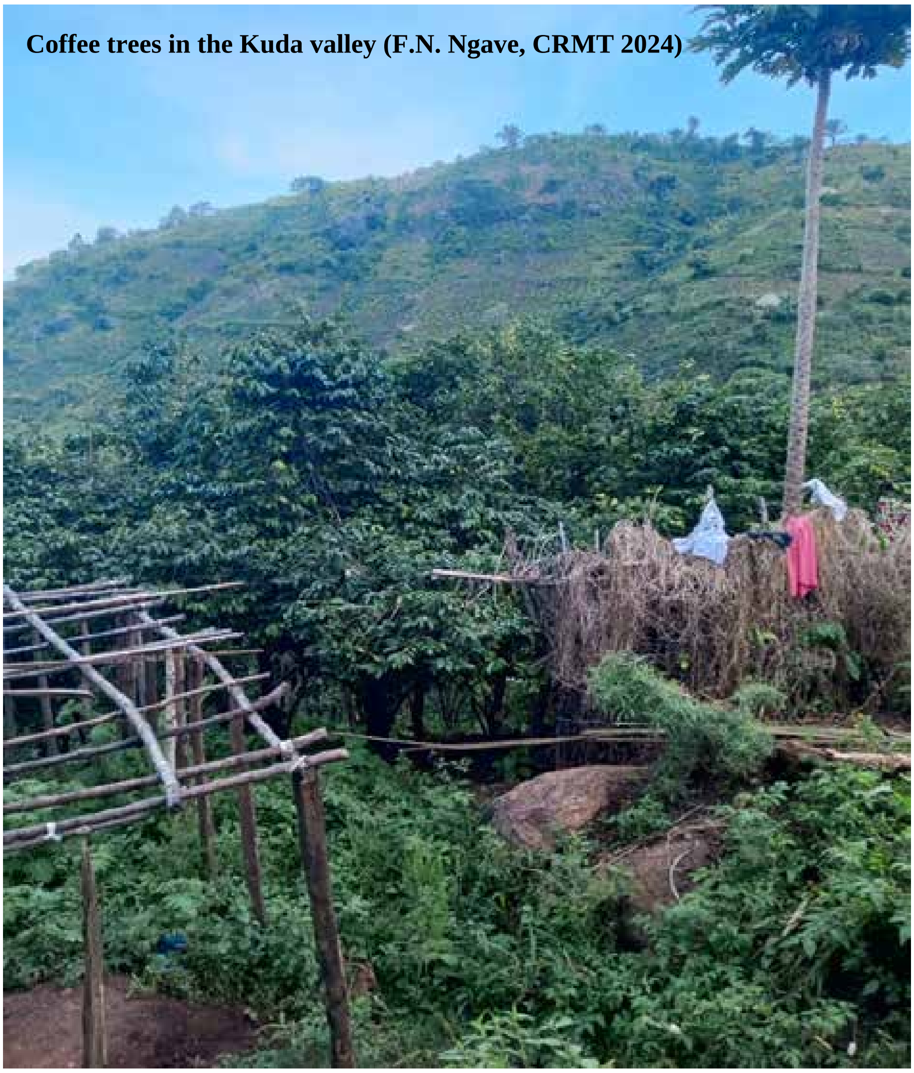

Coffee trees in the Kuda valley (F.N. Ngave, CRMT 2024)

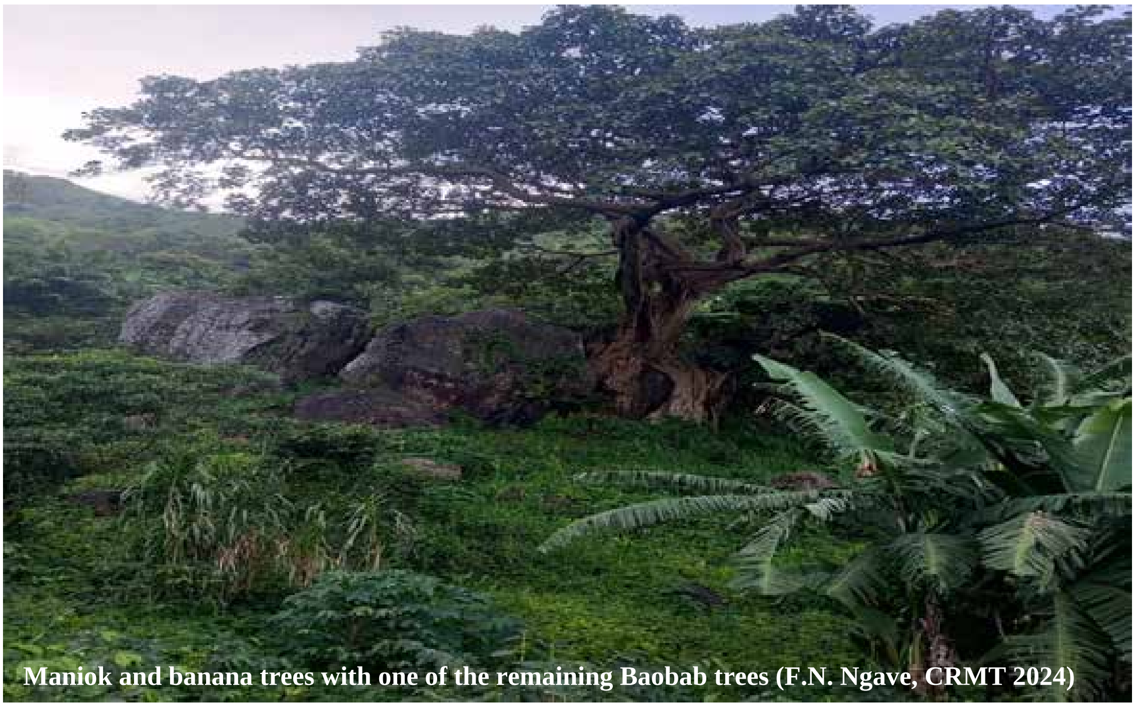
Maniok and banana trees with one of the remaining Baobab trees (F.N. Ngave, CRMT 2024)

Fig S3 Photos of study area taken in 2022-2024 by T.O. Ukety and F.N. Ngave

Table S2 Results of the Rapid Epidemiological Assessment conducted in 2002 as per the site-level ESPEN database (accessed on 14 September 2024) with current Zone and Aire de Santé and APOC CDTI Project

Information extracted from the ESPEN database (accessed 21 February 2025)									ZdS current delineation[3]	AdS current delination[3]	APOC CDTI Project
IU NAME	Location (Village) Name[1]	Survey Year[6]	Method[1]	N examined[1]	N Positive[1]	Nodule Prevalence[2]	Source[1]	Funding			
Rethy	Bessi	2003	Nodule Palpation	30	25	0.83	APOC	APOC TF	Angumu	Bessi	Ituri Sud
Angumu	Dabu-Paju[5]	2003	Nodule Palpation	30	27	0.90	APOC	APOC TF	Angumu	Dabu	Ituri Nord
Angumu	Jupa Lung[5]	2003	Nodule Palpation	37	19	0.51	APOC	APOC TF	Nyarambe	Afoyo	Ituri Nord
Linga	Jupanyaru[5]	2003	Nodule Palpation	50	18	0.36	APOC	APOC TF	Angumu	Kudiweka	Ituri Nord
Angumu	Jupugwoko	2003	Nodule Palpation	30	21	0.70	APOC	APOC TF	Angumu	Langa	Ituri Nord
Rethy	Kanga	2003	Nodule Palpation	30	21	0.70	APOC	APOC TF	Logo	Kanga	Ituri Nord (non CDTI ZdS)
Rethy	Kpagboma	2003	Nodule Palpation	30	11	0.37	APOC	APOC TF	Rethy	Lokpa	Ituri Sud
Linga/ Rethy[4]	Musongwa	2003	Nodule Palpation	49	17	0.35	APOC	APOC TF	Angumu	Musongwa	Ituri Nord
Linga/ Rethy[4]	Ndaru Mus[5]	2003	Nodule Palpation	44	2	0.05	APOC	APOC TF	Angumu	Ndaru	Ituri Nord
Angumu	Ndroy	2003	Nodule Palpation	33	33	1.00	APOC	APOC TF	Logo	Draju	Ituri Nord
Angumu	Thedeja	2003	Nodule Palpation	30	2	0.07	APOC	APOC TF	Logo	Thedeja	Ituri Nord
Angumu	Ukeru-Ban[5]	2003	Nodule Palpation	42	6	0.14	APOC	APOC TF	Nyarambe	Nyalebbe	Ituri Nord (non CDTI ZdS)

AdS Aire de Santé (Health Area), ESPEN: Expanded Special Project for Elimination of Neglected Tropical Diseases (ESPEN) of the WHO African Regional Office, IU Implementation Unit = Zone de Santé as per 2003 Health system delineation, TF Trust Fund, ZdS Zone de Santé (Health Zone), [1] Headings in the ESPEN database modified for clarity, [2] Heading in the ESPEN database modified for clarity and values rounded to two decimals. [3] as per 2014 Division Provinciale Ituri (DPS) delineation and names of Zones de Santé, Aires de Santé and villages, [4] Linga as per ESPEN database is incorrect, the Zone de Santé in 2003 was Rethy. [5] Complete/correct name of village Dabu-Paju is Dabu-Pajuma, of Jupa Lung is Jupalungu, of Jupanyaru is Jupanyandua, of Ndaru Mus is Ndaru Muswa, of Ukeru-Ban is Ukebu Banda (Banda in current DPS village list).
[6] The surveys took place in 2002 but the results were registered by APOC as from 2003

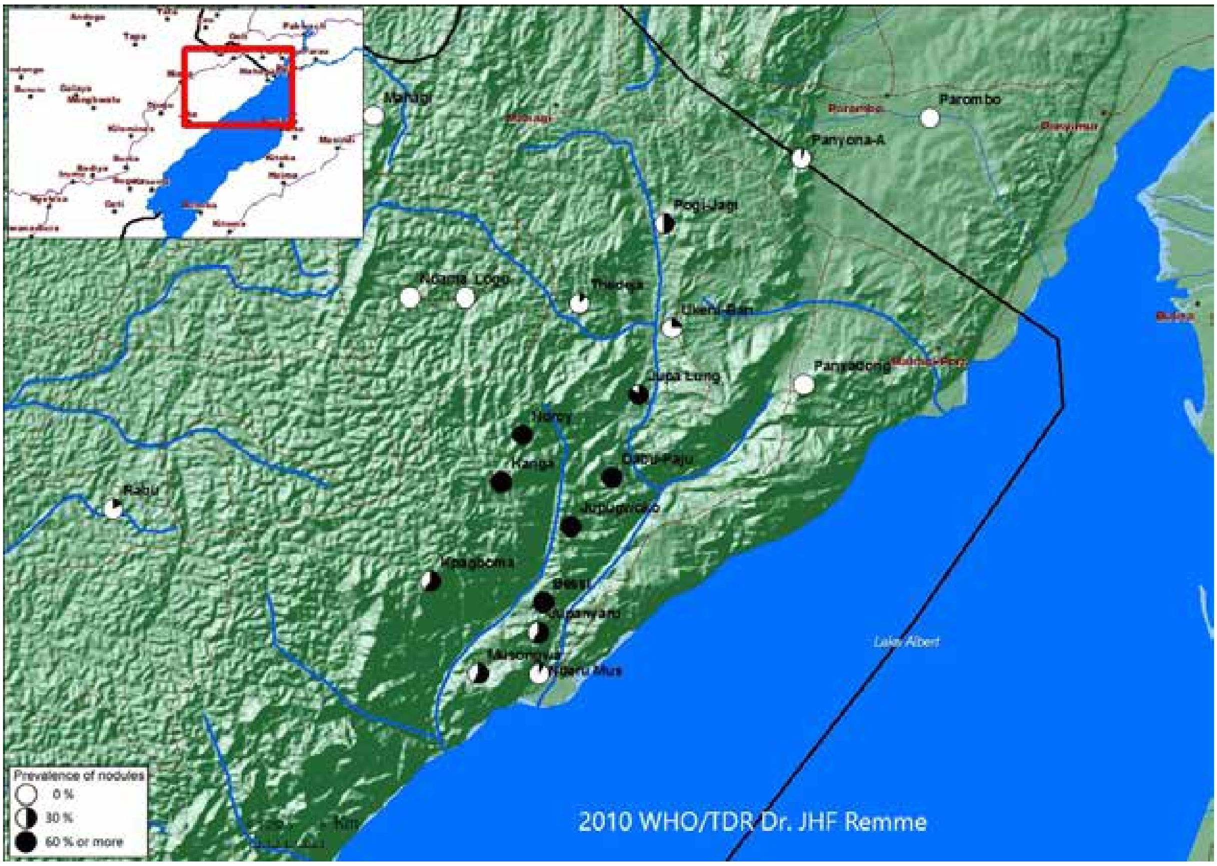

Fig S4 Map of 2002 rapid epidemiological assessment results

Filling of the pies indicates the % of nodule prevalence: white pie 0%, black pie ≥60%. The map was generated in 2010 by Dr. JHF Remme at WHO/TDR to support WHO/TDR discussions internally and with the DRC onchocerciasis control programme on site selection for moxidectin community studies.

Table S3 CRMT capacities, research experience and diseases in the area

**Major equipment at CRMT**	
Clinical evaluation	2 12 lead ECG (with backup)<br>15 Stethoscopes<br>15 Thermoflash<br>15 Digital sphygmomanometer<br>8 Diagnostics kits<br>7 Examinations tables
Ophthalmology	1 Visual acuity chart (illiterates)<br>1 Binocular loupe<br>1 Indirect ophthalmoscope<br>2 Portable slit lamps<br>1 Slit lamps<br>1 Tonometer<br>2 Fundus cameras<br>1 Tono-Pen Avia<br>3 Micro lamp torch<br><br>12 Battery Tonometer<br>1 Battery APC-Electric<br>1 Tonometer Pulser<br>1 Lense trial test box

Laboratory	2 Hematology analyzers(Abacus Junior 5)<br>2 Serum biochemistry analyzers (Cobas c111)<br>2 Electrolyte analyzers<br>2 Clinitek® 50 Urine Chemistry Analyzers<br>7 Olympus 11BF compact inverted binocular microscope with 10x, 40x and 100x objective<br>5 Olympus CX21 FSwith 10x, 40x and 100x objective<br>5 Electronic analytical balances, 320g, 0.1mg<br>1 Benchtop centrifuge (unrefrigerated)<br>3 Cold boxes for sample transport<br>1 Water purification system (Reverse osmosis)<br>2 330 L refrigerator (with out-of-range alarm)<br>3 95 L Freezer to -25 C (with out-of-range alarm)<br>1 145 L Freezer<br>1 112 L Solar freezer<br>2 Sterilisers<br>2 gas stoves
Office equipment	10 Scanner-photocopier-printer<br>1 Scanner<br>2 Printers<br>2 LCD projector<br>33 laptop computers<br>4 desktop computers<br>36 tablets
Transport	3 Landcruiser Toyota<br>1 Hilux Toyota<br>9 Motorcycles
Communication	3 VSAT internet connections
Electricity	Grid electricity (CRMT Rethy)<br>1 Back-up generator (CRMT Rethy)<br>Solar panel electricity (CRMT office Ndrele)<br>1 Solar lighting kit<br>2 Portable generators<br>1 Automatic voltage regulator
**Studies supporting registration/label extension of moxidectin at CRMT Ituri (sponsor)**	
Phase 3 study of moxidectin vs. ivermectin (WHO TDR) [1-4]	
MDGH-MOX-3002 (Medicines Development for Global Health) (data base locked in June 2025)	
MDGH-MOX-3001 (Medicines Development for Global Health) (study ongoing)	
**Other studies conducted in collaboration with CRMT Ituri/past or current CRMT staff**	
Filarial antigenemia and *Loa loa* microfilaremia (Washington University)[5]	
Onchocerciasis associated epilepsy (University of Antwerp, European Research Council (ERC) grant number 671055, project title NSETHIO and VLIRUOS (Flemish Interuniversity Council for University Development Cooperation) [6-16]	
Entomological evaluation [17]	
Parasite genetics [18]	
**Other CRMT collaborations outside DRC**	
National Programme for Onchocerciasis Elimination in Uganda, Vector Control Unit, Ministry of Health, Kampala, Uganda [19]	
**Advanced Research capacity building of past and current CRMT staff**	
Michel Mandro-Ndahura:<br>- One year WHO/TDR Clinical Research Fellowship at Novartis, Switzerland<br>- PhD in 2020, University of Antwerp, Belgium: “Epilepsy in Onchocerciasis endemic villages of Democratic Republic of Congo: epidemiology, clinical and treatment aspects”. [20]	

<table>
<tr><td colspan="2">Maurice Mutro Nigo:
PhD in 2020, University of Basel, Basel, Switzerland, “Schistosomiasis in Eastern Democratic Republic of the Congo: A major neglected healthcare concern”: [21]</td></tr>
<tr><td colspan="2">Jules Ukila Upenjirwoth:
Four months short course in Health Data in Brussels, Université Libre de Bruxelles, Belgium from February to June 2025.</td></tr>
<tr><td colspan="2">Jack Tsulokpa Zawadi:
WHO/TDR Clinical Research Leadership Fellow at DNDi, Geneva, Switzerland, March 2024 to April 2025.</td></tr>
<tr><td colspan="2">Ongoing</td></tr>
<tr><td colspan="2">Pascal Tandele Adroba:
PhD Student since February 2025, l’Institut One Health pour l’Afrique (INOHA), University of Kinshasa, The Democratic Republic of Congo.</td></tr>
<tr><td colspan="2">Gisèle Leming’om Abeditho:
WHO/TDR Clinical Research Leadership Fellow at BioNTech, Mainz, Germany, March 2025 to April 2026.</td></tr>
<tr><td colspan="2">Grants to CRMT</td></tr>
<tr><td colspan="2">European & Developing Countries Clinical Trials Partnership (EDCTP, grant # RIA2017NCT-1843 MoxiMultiDoseMod)</td></tr>
<tr><td colspan="2">Diseases prevalent in the area</td></tr>
<tr><td>Plague
M-Pox
Measles
Onchocerciasis
Schistosomiasis (intestinal)
Lymphatic filariasis
Intestinal helminths
Malaria
Tuberculosis
Leprosy
HIV/AIDs and opportunistic infections
Other sexually transmitted diseases
Hepatitis B
Tropical ulcer (Ulcère phagedenique)
Amoebiasis</td><td>Malnutrition
Diabetes
Hypertension
Epilepsy</td></tr>
</table>

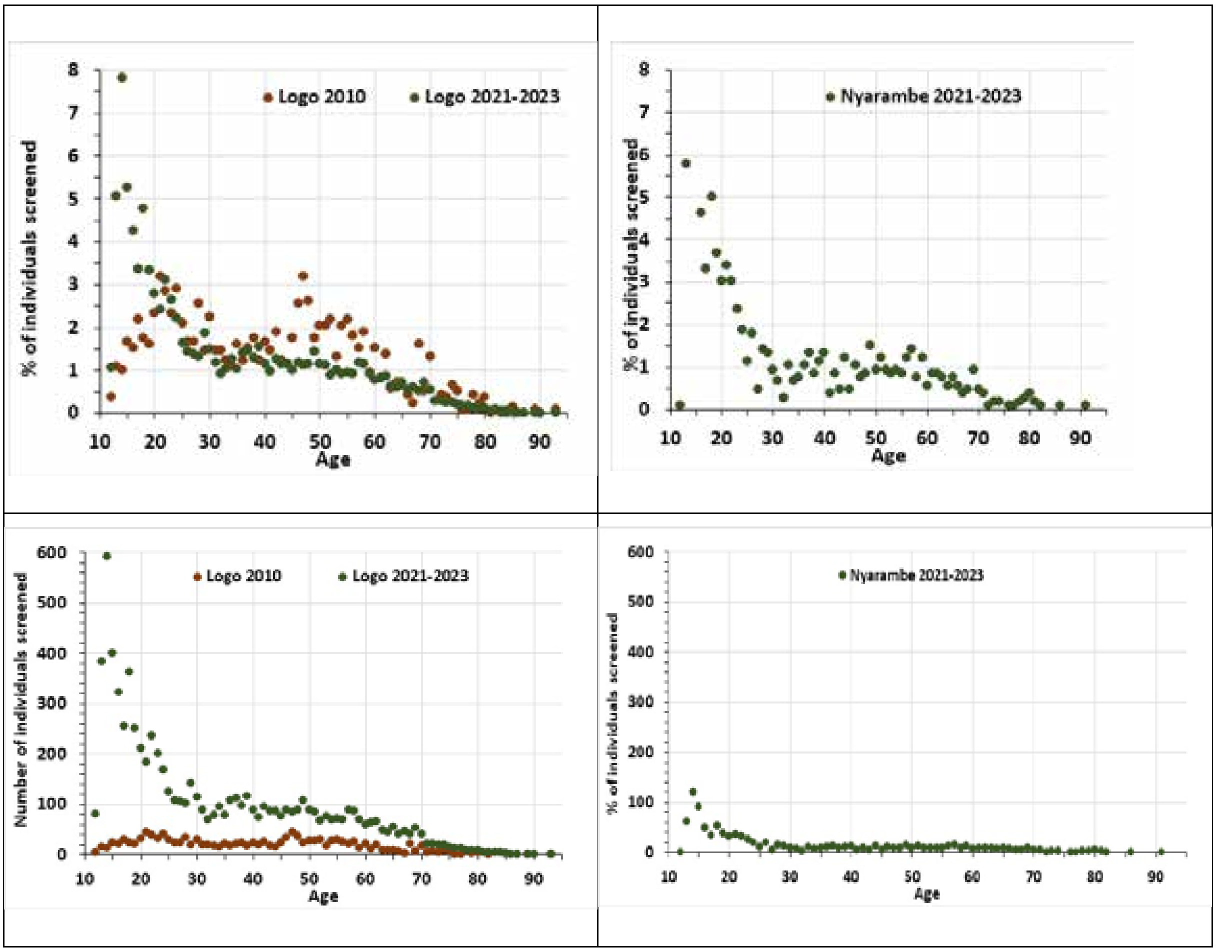

Fig S5 **Percentage and number of individuals screened (lower panel) by age screened in 2010 and 2021-2023 in Zone de Santé (Health Zone) Logo and in 2021-2023 in the Zone de Santé Nyarambe**

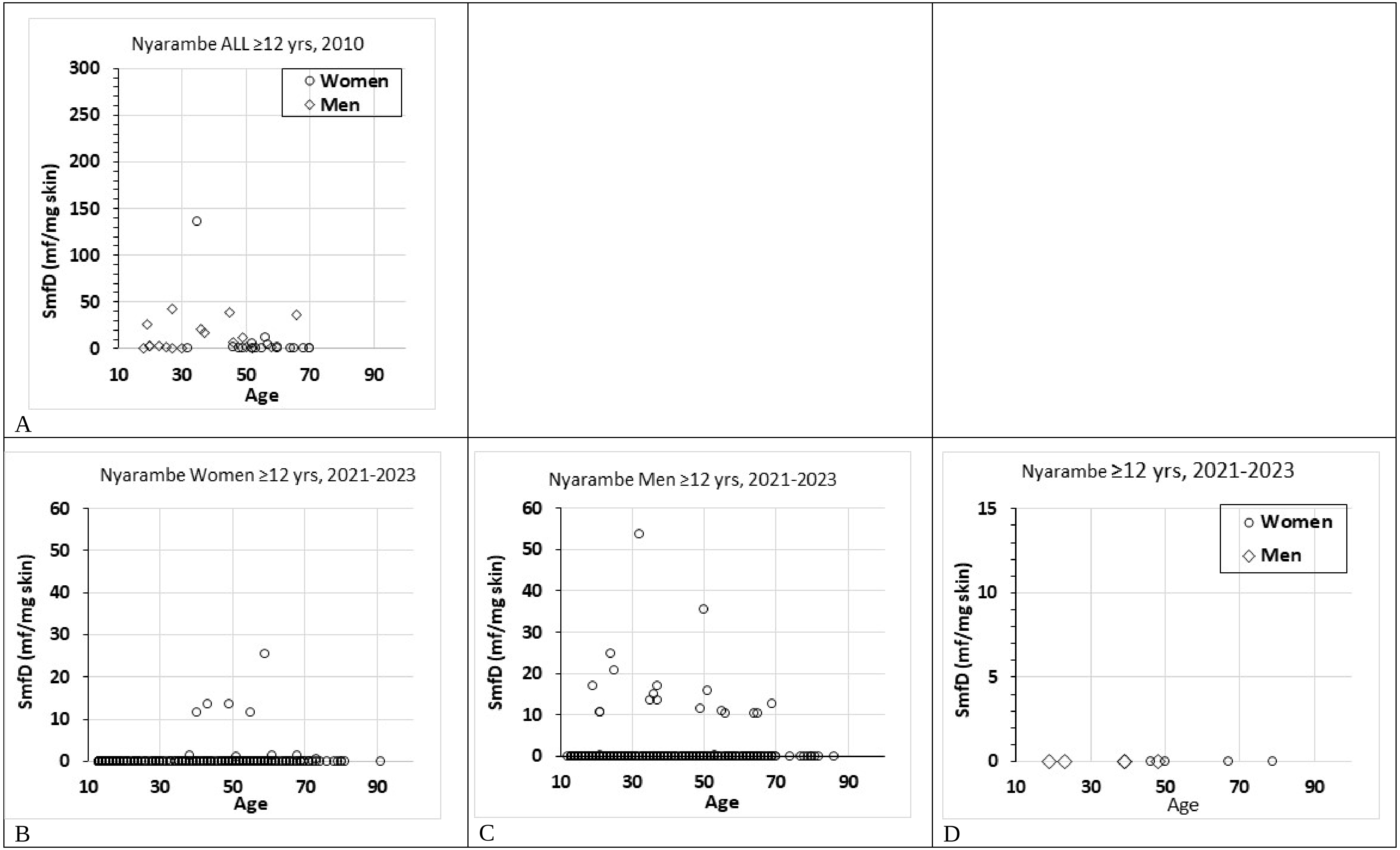

Fig S6 **Skin microfilariae density among all women and men (A) screened in 2010, and women (B) and men (C) screened in 2021-2023 who had no prior ivermectin treatment and (D) women and men reporting ivermectin treatment between 2 and 1800 days before screening in the Zone de Santé (Health Zone) Nyarambe**

SmfD skin microfilariae density, mf microfilariae, A n women = 18, n men = 18, B n women=443, C n men = 604, D n women =4, men= 5

Table S4 Number of participants without prior IVM treatment screened in 2010 and 2021-2023 by age group, gender and skin microfilariae density category by Zone de Santé (Health Zone)

ZdS	Period	Age	Sex		0		>0-<5		≥5-<10		≥10-<20		≥20-<30		≥30-<40		≥40-<50		≥50-<60		≥60-<80		≥80	
		(Years)		N	n	%	n	%	n	%	n	%	n	%	n	%	n	%	n	%	n	%	n	%
LOGO	2010	All	All	1373	324	**23.6**	285	**20.8**	116	**8.4**	132	**9.6**	112	**8.2**	92	**6.7**	71	**5.2**	50	**3.6**	87	**6.3**	104	**7.6**
LOGO	2010	≥18	All	1265	295	**23.3**	251	**19.8**	109	**8.6**	121	**9.6**	101	**8.0**	87	**6.9**	66	**5.2**	50	**4.0**	81	**6.4**	104	**8.2**
LOGO	2010	≥18	F	471	97	**20.6**	105	**22.3**	43	**9.1**	47	**10.0**	42	**8.9**	31	**6.6**	25	**5.3**	20	**4.2**	25	**5.3**	36	**7.6**
LOGO	2010	≥18	M	794	198	**24.9**	146	**18.4**	66	**8.3**	74	**9.3**	59	**7.4**	56	**7.1**	41	**5.2**	30	**3.8**	56	**7.1**	68	**8.6**
LOGO	2010	12-17	All	108	29	**26.9**	34	**31.5**	7	**6.5**	11	**10.2**	11	**10.2**	5	**4.6**	5	**4.6**			6	**5.6**		
LOGO	2010	12-17	F	35	10	**28.6**	15	**42.9**	4	**11.4**	3	**8.6**	3	**8.6**										
LOGO	2010	12-17	M	73	19	**26.0**	19	**26.0**	3	**4.1**	8	**11.0**	8	**11.0**	5	**6.8**	5	**6.8**			6	**8.2**		
LOGO	21-23	All	All	7547	6922	**91.7**	269	**3.6**	52	**0.7**	196	**2.6**	38	**0.5**	28	**0.4**	10	**0.1**	15	**0.2**	8	**0.1**	9	**0.1**
LOGO	21-23	≥18	All	5517	4956	**89.8**	232	**4.2**	47	**0.9**	178	**3.2**	37	**0.7**	27	**0.5**	10	**0.2**	15	**0.3**	6	**0.1**	9	**0.2**
LOGO	21-23	≥18	F	2519	2307	**91.6**	96	**3.8**	22	**0.9**	62	**2.5**	11	**0.4**	11	**0.4**	3	**0.1**	2	**0.1**	2	**0.1**	3	**0.1**
LOGO	21-23	≥18	M	2998	2649	**88.4**	136	**4.5**	25	**0.8**	116	**3.9**	26	**0.9**	16	**0.5**	7	**0.2**	13	**0.4**	4	**0.1**	6	**0.2**
LOGO	21-23	12-17	All	2030	1966	**96.8**	37	**1.8**	5	**0.2**	18	**0.9**	1	**0.0**	1	**0.0**					2	**0.1**		
LOGO	21-23	12-17	F	1006	979	**97.3**	13	**1.3**	3	**0.3**	11	**1.1**												
LOGO	21-23	12-17	M	1024	987	**96.4**	24	**2.3**	2	**0.2**	7	**0.7**	1	**0.1**	1	**0.1**					2	**0.2**		
Nya	2010	All	All	36	10	**27.8**	15	**41.7**	2	**5.6**	3	**8.3**	2	**5.6**	2	**5.6**	1	**2.8**					1	**2.8**
Nya	2010	≥18	All	36	10	**27.8**	15	**41.7**	2	**5.6**	3	**8.3**	2	**5.6**	2	**5.6**	1	**2.8**					1	**2.8**
Nya	2010	≥18	F	18	7	**38.9**	8	**44.4**	1	**5.6**	1	**5.6**											1	**5.6**
Nya	2010	≥18	M	18	3	**16.7**	7	**38.9**	1	**5.6**	2	**11.1**	2	**11.1**	2	**11.1**	1	**5.6**						
Nya	21-23	All	All	1047	1017	**97.1**	7	**0.7**			18	**1.7**	3	**0.3**	1	**0.1**			1	**0.1**				
Nya	21-23	≥18	All	690	660	**95.7**	7	**1.0**			18	**2.6**	3	**0.4**	1	**0.1**			1	**0.1**				
Nya	21-23	≥18	F	270	260	**96.3**	5	**1.9**			4	**1.5**	1	**0.4**										
Nya	21-23	≥18	M	420	400	**95.2**	2	**0.5**			14	**3.3**	2	**0.5**	1	**0.2**			1	**0.2**				
Nya	21-23	12-17		357	357	**100**																		
Nya	21-23	12-17	F	173	173	**100**																		
Nya	21-23	12-17	M	184	184	**100**																		

Nya Nyarambe, 21-23 2021-2023

Table S5 Number of adults and adolescents without prior ivermectin treatment screened in 2010 and in 2021-2023 with and without detectable SmfD

			Period	2010	2010	2010	2010	2010	2010	2021-2023	2021-2023	2021-2023	2021-2023	2021-2023	2021-2023
			Age	≥18	≥18	12-17	12-17	Total	Total	≥18	≥18	12-17	12-17	Total	Total
			SmfD	0	>0	0	>0		%>0	0	>0	0	>0		%>0
River basin	ZdS	AdS	Village												
Awo	LOGO	THEDEJA	ANG'AL UNEN							10		1		11	0.0
Awo	LOGO	THEDEJA	DRAYI							79	6	14		99	6.1
Awo	LOGO	THEDEJA	JUPAJAZA							84	3	9		96	3.1
Awo	LOGO	THEDEJA	JUPUYURU							20		4		24	0.0
Awo	LOGO	THEDEJA	PALEY							42		3		45	0.0
Awo	LOGO	THEDEJA	THEDEJA 2							102	3	26		131	2.3
Awo	LOGO	THEDEJA	UCUDO							36	4	4		44	9.1
Awo	LOGO	THEDEJA	URYANG							70	5	10		85	5.9
Awo	LOGO	THEDEJA	VUNA							25	2	2		29	6.9
Awo	LOGO	THEDEJA	WIRAA							57	2	15		74	2.7
Awo	LOGO	THEDEJA	WILOO							42	3	5		50	6.0
Awo	LOGO	THEDEJA	Thecer							13		1		14	0.0
Awo	LOGO	THEDEJA	THEDÉJÀ 1							88	2	16		106	1.9
Awo	LOGO	THEDEJA	MADI KAKA							47	2	2		51	3.9
Awo	Nyarambe	KPANYI	Pacung		2			2	100.0						
Awo	Nyarambe	LELO	Awora		4			4	100.0						
Awo	Nyarambe	LELO	Mbraze		1			1	100.0						
Kuda	LOGO	AMBERE	JUPUKELO	2	9		3	14	85.7						
Kuda	LOGO	BUU	BUU MISSION	1				1	0.0						
Kuda	LOGO	BUU	MADI		3			3	100.0						
Kuda	LOGO	DRAJU	DRAJU	15	46	1	4	66	75.8	293	18	163		474	3.8
Kuda	LOGO	DRAJU	JUPADROGO	9	17			26	65.4	141	13	38	1	193	7.3
Kuda	LOGO	DRAJU	KONDU		4			4	100.0	69	5	31	1	106	5.7
Kuda	LOGO	DRAJU	KPANA	47	192	12	32	283	79.2	152	28	122	5	307	10.7

			Period	2010	2010	2010	2010	2010	2010	2021-2023	2021-2023	2021-2023	2021-2023	2021-2023	2021-2023
			Age	≥18	≥18	12-17	12-17	Total	Total	≥18	≥18	12-17	12-17	Total	Total
			SmfD	0	>0	0	>0		%>0	0	>0	0	>0		%>0
River basin	ZdS	AdS	Village												
Kuda	LOGO	DRAJU	MAKALA	1	4		2	7	85.7	123	16	72	4	215	9.3
Kuda	LOGO	DRAJU	MBESI		7			7	100.0	71	27	30	7	135	25.2
Kuda	LOGO	DRAJU	NDROY	5	60		8	73	93.2	44	20	39	3	106	21.7
Kuda	LOGO	DRAJU	NGBUNGBU	24	105	4	3	136	79.4	97	25	24	1	147	17.7
Kuda	LOGO	DRAJU	NYODU							641	42	263	4	950	4.8
Kuda	LOGO	DRAJU	NZURU		12			12	100.0	102	11	51	3	167	8.4
Kuda	LOGO	DRAJU	RUJU	16	116	2	10	144	87.5	112	37	64	5	218	19.3
Kuda	LOGO	DRAJU	UMULO	79	235	2	8	324	75.0	219	35	70		324	10.8
Kuda	LOGO	DRAJU	YAU	23	40	1	3	67	64.2	415	47	207	4	673	7.6
Kuda	LOGO	JUPAHOY	JUPAHOY		1			1	100.0						
Kuda	LOGO	JUPAHOY	JUPALEBE	1	2		1	4	75.0						
Kuda	LOGO	JUPAHOY	PAMUNDU	3				3	0.0						
Kuda	LOGO	KANGA	JABI	2	14			16	87.5	314	44	45	1	404	11.1
Kuda	LOGO	KANGA	JUPARIMA	3	21		1	25	88.0	389	49	237	7	682	8.2
Kuda	LOGO	KANGA	LOO		2			2	100.0	391	40	134	5	570	7.9
Kuda	LOGO	NDRELE	AWURA	10				10	0.0						
Kuda	LOGO	ULYEKO	JUPALIRI GULUKPA	3				3	0.0						
Kuda	LOGO	ULYEKO	ULYEKO	9	3	1	1	14	28.6						
Kuda	Nyarambe	KPANYI	Gbii							32	4	20		56	7.1
Kuda	Nyarambe	KPANYI	Jupafoyo							83	1	53		137	0.7
Kuda	Nyarambe	KPANYI	Jupajalbonyo							37		3		40	0.0
Kuda	Nyarambe	KPANYI	Jupanyamoro							117	5	76		198	2.5
Kuda	Nyarambe	KPANYI	jupasugu							98	4	57		159	2.5
Kuda	Nyarambe	KPANYI	Jupawalu							68		53		121	0.0
Kuda	Nyarambe	KPANYI	Jupawegi							3				3	0.0

			Period	2010	2010	2010	2010	2010	2010	2021-2023	2021-2023	2021-2023	2021-2023	2021-2023	2021-2023
			Age	≥18	≥18	12-17	12-17	Total	Total	≥18	≥18	12-17	12-17	Total	Total
			SmfD	0	>0	0	>0		%>0	0	>0	0	>0		%>0
River basin	ZdS	AdS	Village												
Kuda	Nyarambe	KPANYI	Jupudero 1							59	2	31		92	2.2
Kuda	Nyarambe	KPANYI	jupudero 2	5	16			21	76.2	14		3		17	0.0
Kuda	Nyarambe	KPANYI	Jupujanga							26	3	8		37	8.1
Kuda	Nyarambe	KPANYI	Jupuvuga							89	10	45		144	6.9
Kuda	Nyarambe	KPANYI	Kpanyi	1				1	0.0	34	1	8		43	2.3
Kuda	Nyarambe	KPANYI	Umulo 1	4	3			7	42.9						
Lebu	LOGO	KANGA	CUCU	1	5			6	83.3						
Lebu	LOGO	KANGA	KANGA	35	53	6	3	97	57.7	279	19	90	6	394	6.3
Lebu	LOGO	KANGA	MOO		6			6	100.0	172	32	89	5	298	12.4
Lebu	LOGO	KANGA	RAA	2				2	0.0						
Lebu	LOGO	KANGA	DYAMBU	1	6			7	85.7	217	21	85	2	325	7.1
Lebu	LOGO	WALLA	BUGO	2	6			8	75.0						
Lebu	LOGO	WALLA	JUPANJAYA	1	1			2	50.0						

AdS Aire de Santé, ZdS Zone de Santé

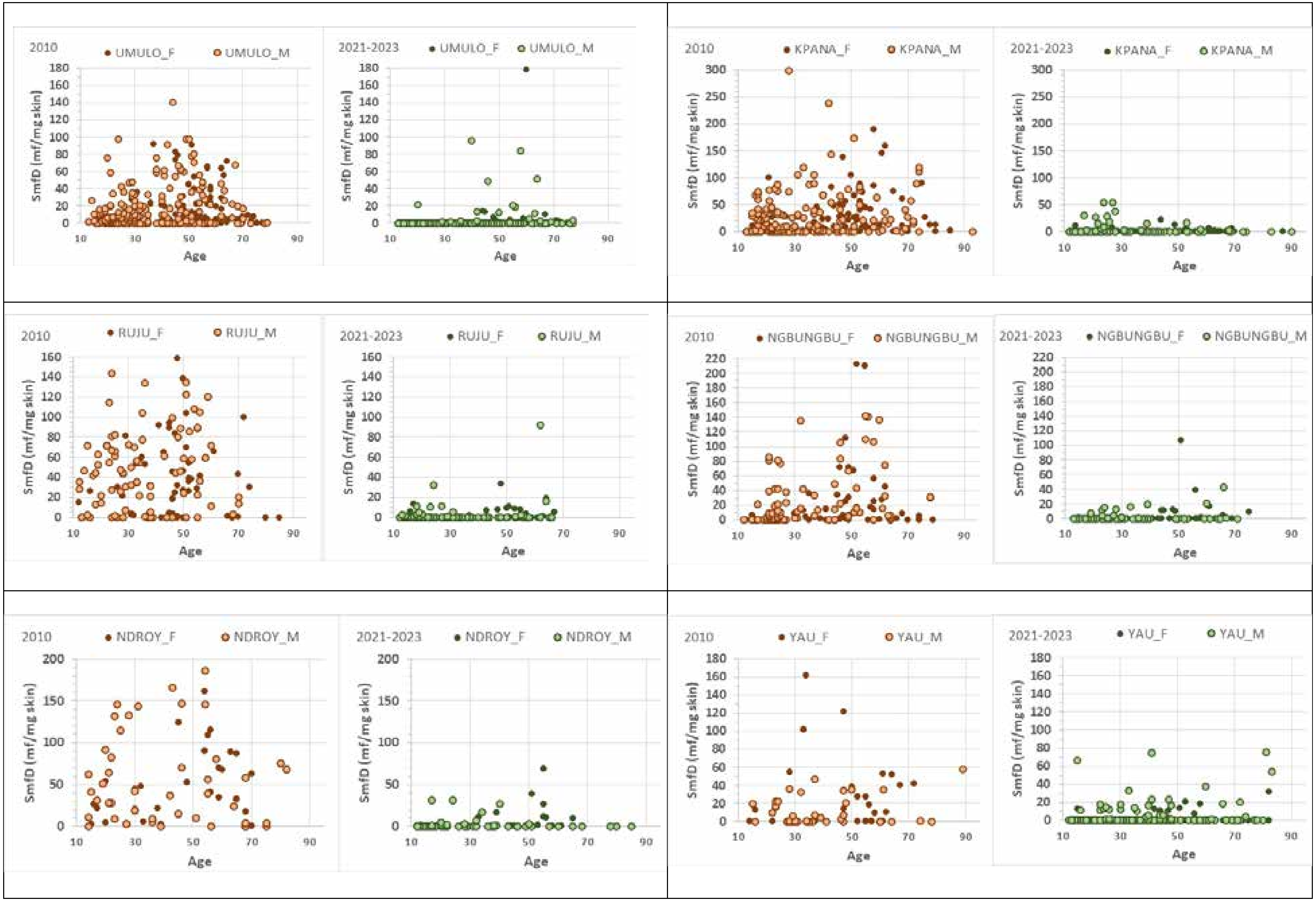
2010
UMULO_F
UMULO_M
2021-2023
KPANA_F
KPANA_M
RUJU_F
RUJU_M
NGBUNGBU_F
NGBUNGBU_M
NDROY_F
NDROY_M
YAU_F
YAU_M
SmfD (mf/mg skin)
Age

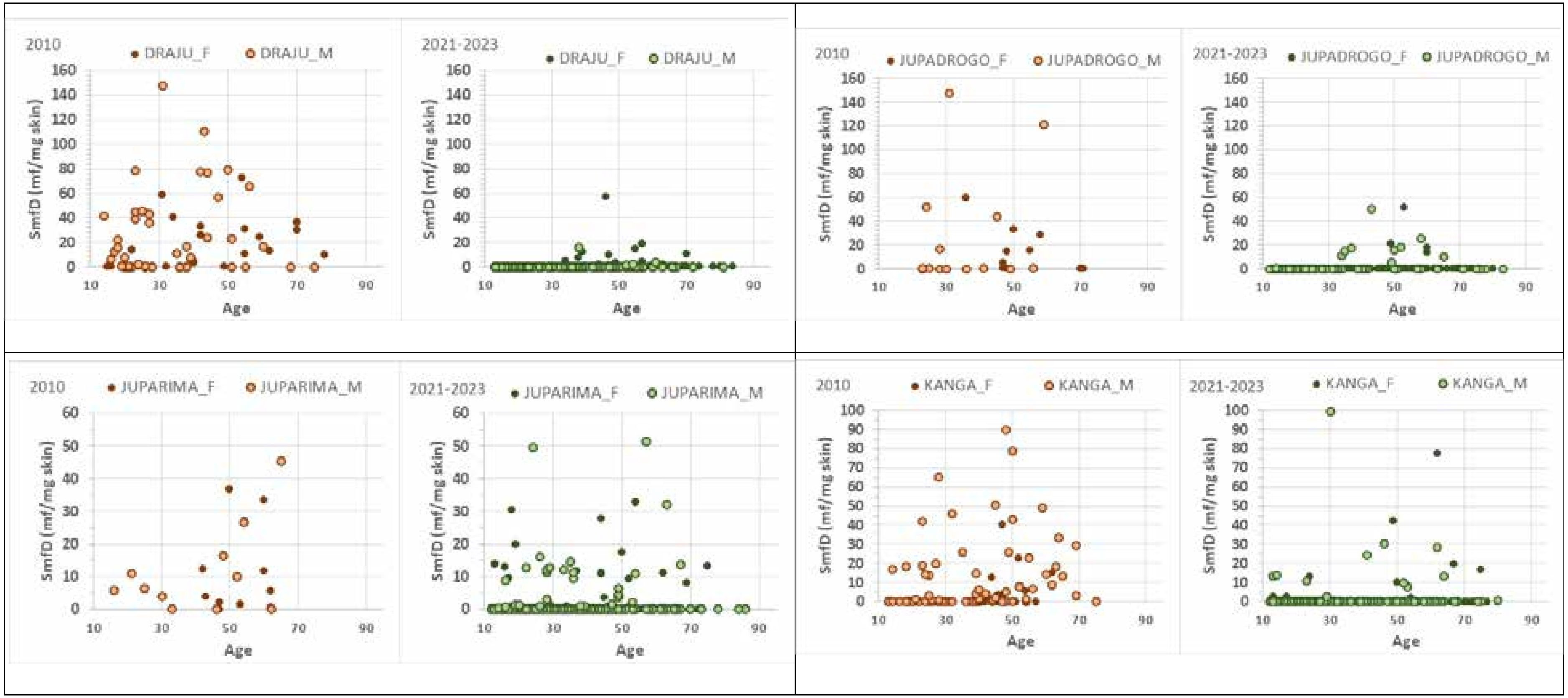

Fig S7 **Skin microfilariae density among volunteers in the 10 villages where at least 20 individuals without prior ivermectin treatment were screened in 2010 and in 2021-2023 by age and gender**

_F women, _M men

In 2010, the number of adults screened in each village ranged from 24 to 314, the number of adolescents ranged from 0 (Jupadrogo) to 44 with less than 10 adolescents screened in Yau, Draju, Jupadrogo, Ndroy, Ngbungbu, Juparima and Kanga. In 2021-2023, the number of adults ranged from 64 to 462 and that of adolescents from 25 to 244. More details are provided in Additional File 1 Table S5

Table S6 Descriptive statistics of SmfD for mf positive individuals without prior ivermectin treatment screened in 2010 and in 2021-2023

			**Period**	**2010**	**2010**	**2010**	**2010**	**2010**	**2021-2023**	**2021-2023**	**2021-2023**	**2021-2023**	**2021-2023**
			**SmfD**	**>0**	**>0**	**>0**	**>0**	**>0**	**>0**	**>0**	**>0**	**>0**	**>0**
**Basin**			**Village**	**n**	**AM**	**SD**	**Min**	**Max**	**n**	**AM**	**SD**	**Min**	**Max**
Awo	LOGO	THEDEJA	DRAYI						6	11.1	11.89	0.8	33.3
Awo	LOGO	THEDEJA	JUPAJAZA						3	1.7	1.40	0.4	3.1
Awo	LOGO	THEDEJA	THEDEJA 2						3	18.8	9.47	10.4	29.0
Awo	LOGO	THEDEJA	UCUDO						4	5.3	8.67	0.3	18.2
Awo	LOGO	THEDEJA	URYANG						5	11.5	12.19	1.3	26.5
Awo	LOGO	THEDEJA	VUNA						2	6.5	6.60	1.8	11.1
Awo	LOGO	THEDEJA	WIRAA						2	14.6	18.30	1.7	27.5
Awo	LOGO	THEDEJA	WILOO						3	58.3	84.09	1.2	154.8
Awo	LOGO	THEDEJA	THEDÉJÀ 1						2	0.4	0.05	0.3	0.4
Awo	LOGO	THEDEJA	MADI KAKA						2	3.4	4.31	0.4	6.5
Awo	Nyarambe	KPANYI	Pacung	2	3.8	2.57	2.0	5.6					
Awo	Nyarambe	LELO	Awora	4	3.2	5.93	0.1	12.1					
Awo	Nyarambe	LELO	Mbraze	1	1.4		1.4	1.4					
Kuda	LOGO	AMBERE	JUPUKELO	12	15.7	15.87	1.4	40.0					
Kuda	LOGO	BUU	MADI	3	38.2	37.18	3.5	77.5					
Kuda	LOGO	DRAJU	DRAJU	50	30.8	31.44	0.2	147.5	18	9.6	13.23	0.3	57.6
Kuda	LOGO	DRAJU	JUPADROGO	17	31.8	43.41	0.2	147.9	14	19.3	14.65	0.2	51.3
Kuda	LOGO	DRAJU	KONDU	4	29.8	20.82	2.2	47.4	6	16.8	16.05	0.3	47.6
Kuda	LOGO	DRAJU	KPANA	224	32.6	42.40	0.1	299.4	33	12.4	14.67	0.1	54.6
Kuda	LOGO	DRAJU	MAKALA	6	12.2	16.05	0.1	39.4	20	10.2	11.39	0.4	34.0
Kuda	LOGO	DRAJU	MBESI	7	61.3	31.04	13.6	119.3	34	11.2	18.00	0.1	64.5
Kuda	LOGO	DRAJU	NDROY	68	56.6	49.24	0.2	186.2	23	14.2	16.66	0.6	69.1
Kuda	LOGO	DRAJU	NGBUNGBU	108	31.3	43.25	0.1	212.2	26	15.0	21.56	0.3	106.4
Kuda	LOGO	DRAJU	NYODU						46	10.9	11.24	0.1	49.2
Kuda	LOGO	DRAJU	NZURU	12	42.7	22.72	5.2	79.2	14	10.6	10.97	0.3	39.6
Kuda	LOGO	DRAJU	RUJU	126	48.2	36.96	0.1	158.4	42	8.5	15.33	0.1	92.3

			**Period**	**2010**	**2010**	**2010**	**2010**	**2010**	**2021-2023**	**2021-2023**	**2021-2023**	**2021-2023**	**2021-2023**
			**SmfD**	**>0**	**>0**	**>0**	**>0**	**>0**	**>0**	**>0**	**>0**	**>0**	**>0**
**Basin**			**Village**	**n**	**AM**	**SD**	**Min**	**Max**	**n**	**AM**	**SD**	**Min**	**Max**
Kuda	LOGO	DRAJU	UMULO	243	20.8	24.95	0.1	140.5	35	18.4	35.75	0.1	177.8
Kuda	LOGO	DRAJU	YAU	43	28.5	33.22	0.1	161.3	51	15.3	18.00	0.1	75.9
Kuda	LOGO	JUPAHOY	JUPAHOY	1	0.2	#DIV/0!	0.2	0.2					
Kuda	LOGO	JUPAHOY	JUPALEBE	3	5.8	7.80	0.2	14.7					
Kuda	LOGO	KANGA	JABI	14	50.9	60.13	2.5	200.8	45	18.6	19.80	0.2	99.2
Kuda	LOGO	KANGA	JUPARIMA	22	24.2	31.01	0.2	132.3	56	10.0	11.44	0.1	51.4
Kuda	LOGO	KANGA	LOO	2	54.5	8.74	48.3	60.7	45	12.2	12.28	0.1	58.3
Kuda	LOGO	ULYEKO	ULYEKO	4	6.3	6.25	0.2	14.3					
Kuda	Nyarambe	KPANYI	Gbii						4	22.7	20.81	10.5	53.8
Kuda	Nyarambe	KPANYI	Jupafoyo						1	1.3	#DIV/0!	1.3	1.3
Kuda	Nyarambe	KPANYI	Jupanyamoro						5	14.7	12.83	0.4	35.7
Kuda	Nyarambe	KPANYI	Jupasugu						4	8.1	4.55	1.3	10.6
Kuda	Nyarambe	KPANYI	Jupudero 1						2	17.7	9.96	10.6	24.7
Kuda	Nyarambe	KPANYI	jupudero 2	16	17.5	33.64	0.3	135.6					
Kuda	Nyarambe	KPANYI	Jupujanga						3	16.6	7.73	10.9	25.4
Kuda	Nyarambe	KPANYI	Jupuvuga						10	9.8	8.18	0.2	20.7
Kuda	Nyarambe	KPANYI	Kpanyi						1	13.6	#DIV/0!	13.6	13.6
Kuda	Nyarambe	KPANYI	Umulo 1	3	26.0	19.24	3.8	38.4					
Lebu	LOGO	KANGA	CUCU	5	28.5	26.47	0.1	59.2					
Lebu	LOGO	KANGA	KANGA	56	16.6	20.56	0.1	89.7	25	17.7	24.13	0.1	99.1
Lebu	LOGO	KANGA	MOO	6	19.4	18.59	0.1	38.5	37	9.3	19.07	0.1	94.9
Lebu	LOGO	KANGA	DYAMBU	6	34.6	29.69	3.7	76.3	23	13.9	14.38	0.6	50.1
Lebu	LOGO	WALLA	BUGO	6	28.8	32.15	1.3	91.1					
Lebu	LOGO	WALLA	JUPANJAYA	1	37.7		37.7	37.7					

AM: Arithmetic mean, Nya Nyarambe, SD Standard deviation

Table S7 Publicly available data on onchocerciasis prevalence in and around the Zone de Santés Nyarambe and Logo

Year	Method	Results	Source / reference
2002-2003	Nodule palpation	Nodule prevalence 0.07 to 1.0, (for details see Additional File 1 Table S2)<br>Nodule prevalence range would correspond to an mf-positivity prevalence of 10 – 100 % as per [22]	WHO ESPEN site level onchocerciasis database accessed 21 Feb 2025
2015	Skin snips<br><br><br><br><br>Ov16 IG4 - ELISA	ZdS Nyarambe- village Angaba, 0/302 mf positive<br>ZdS Rethy – village Djupanjana, 0/118 mf positive (based on the GPS coordinates provided, this village is Djupanjaya in the ZdS Logo)<br>ZdS Angumu – village Madi, 0/210 mf positive<br>ZdS Mahagi – village Tilal, 0/311 mf positive<br>ZdS Nyarambe- village Angaba, 1/302 positive<br>ZdS Rethy – village Djupanjana, 32/118 positive (based on the GPS coordinates provided, this village is Djupanjaya in the ZdS Logo)<br>ZdS Angumu – village Madi, 11/210 positive<br>ZdS Mahagi – village Tilal, 1/311 positive	WHO ESPEN https://espen.afro.who.int/ site level onchocerciasis database<br>accessed 21 Feb 2025
2015	 Ov16 IG4 RDT<br>Microscopy*<br><br>Ov16 IG4 RDT<br>Microscopy*	ZdS Logo – village Draju<br>Persons with epilepsy: 30/59 (51%) Ov16 IG4 positive, Controls: 14/65 (22%) Ov16 IG4 positive<br>Persons with epilepsy: 33/59 (56%) mf positive, Controls: 17/65 (26%) mf positive<br>ZdS Rethy – village Rassia<br>Persons with epilepsy: 21/49 (43%) Ov16 IG4 positive, Controls 9/45 (20%) Ov16 IG4 positive<br>Persons with epilepsy: 17/49 (35%) mf positive, Controls: 9/45 (20%) mf positive	[23]
2016	Ov16 IG4 RDT	Random household survey in<br>ZdS Logo – AdS Draju, villages Nzuru, Ruju, Kpana, Umulo, Makala, Ndroy, Mbesi, Yau, Draju, Kondu, Nyodu, Jupadrogo: 155/433 (35.8%) OV16 IG4 positive<br>ZdS Logo – AdS Kanga, villages Kanga, Juparima, Jabi, Djambu, Cucu, Wiloo, Raa, Nguu: 123/479 (25.7%) OV16 IG4 positive	[24]
2017	 Ov16 IG4 RDT<br>Microscopy*	Persons with epilepsy<br>ZdS Logo – villages Draju, Kanga, Wala, Ulyeko and Thedeja<br>65/90 (72.2%) Ov16 IG4 positive<br>67/90 (74.4%) mf positive	[14]
2018	 Ov16 IG4 RDT<br>Microscopy*	Persons with epilepsy<br>ZdS Logo – villages Draju, Kanga, Wala, Ulyeko and Thedeja<br>144/197 (73%) Ov16 IG4 positive<br>141/197 (71.6%) mf positive	[13]

AdS Aire de Santé, ELISA Enzyme-linked immunosorbent assay, ESPEN Expanded Special Project for Elimination of Neglected Tropical Diseases in the WHO Regional Office for Africa, RDT Rapid Diagnostic Test SD Bioline, ZdS Zone de Santé, * two skin snips

Table S8 Prevalence of individuals with >0 *O. volvulus* microfilariae detected in the two iliac crests by village

		2010	2010	2010	2010	2021-2023	2021-2023	2021-2023	2021-2023	2021-2023	2021-2023	2021-2023	2021-2023
**Age group**		**≥18**	**≥18**	**12-17**	**12-17**	**≥18**	**≥18**	**≥18**	**≥18**	**12-17**	**12-17**	**12-17**	**12-17**
**Prior IVM Tx**		**No**	**No**	**No**	**No**	**No**	**No**	**Yes**	**Yes**	**No**	**No**	**Yes**	**Yes**
**Zone de Santé**	**Village**	**N**	**% >0**	**N**	**% >0**	**N**	**% >0**	**N**	**% >0**	**N**	**% >0**	**N**	**% >0**
LOGO	ANG'AL UNEN	0		0		10	0	0		1	0	0	
	AWURA	10	0	0		0		0		0		0	
	BUGO	8	75.0	0		0		0		0		0	
	BUU MISSION	1	0	0		0		0		0		0	
	CUCU	6	83.3	0		0		0		0		0	
	DRAJU	61	75.4	5	80.0	311	5.5	2	50.0	163	0	1	0
	DRAY	0		0		85	7.1	0		14	0	0	
	DYAMBU	7	85.7	0		238	8.0	0		87	2.3	0	
	JABI	16	87.5	0		358	12.0	3	33.3	46	2.2	0	
	JUPADROGO	26	65.4	0		154	8.4	1	0	39	0	0	
	JUPAHOY	1	100	0		0		0		0		0	
	JUPAJAZA	0		0		87	2.3	0		9	0	0	
	JUPALEBE	3	66.7	1	0	0		0		0		0	
	JUPALIRI GULUKPA	3	0	0		0		0		0		0	
	JUPANJAYA	2	50.0	0		0		0		0		0	
	JUPARIMA	24	87.5	1	100	438	10.3	0		244	2.5	0	
	JUPUKELO	11	81.8	3	100	0		0		0		0	
	JUPUYURU	0		0		20	0	0		4	0	0	
	KANGA	88	59.1	9	33.3	298	6.4	1	0	96	6.3	2	0
	KONDU	4	100	0		74	5.4	0		32	3.1	0	

		2010	2010	2010	2010	2021-2023	2021-2023	2021-2023	2021-2023	2021-2023	2021-2023	2021-2023	2021-2023
**Age group**		**≥18**	**≥18**	**12-17**	**12-17**	**≥18**	**≥18**	**≥18**	**≥18**	**12-17**	**12-17**	**12-17**	**12-17**
**Prior IVM Tx**		**No**	**No**	**No**	**No**	**No**	**No**	**Yes**	**Yes**	**No**	**No**	**Yes**	**Yes**
**Zone de Santé**	**Village**	**N**	**% >0**	**N**	**% >0**	**N**	**% >0**	**N**	**% >0**	**N**	**% >0**	**N**	**% >0**
	KPANA	239	77.0	44	61.4	180	15.0	2	0	127	3.1	0	
	LOO	2	100	0		431	9.0	7	0	139	2.2	1	0
	MADI	3	100	0		0		0		0		0	
	MADI KAKA	0		0		49	4.1	0		2	0	0	
	MAKALA	5	80.0	2	100	139	10.8	0		76	5.3	0	
	MBESI	7	100	0		98	25.5	0		37	16.2	0	
	MOO	6	100	0		204	14.2	1	0	94	5.3	0	
	NDROY	65	90.8	8	100	64	29.7	3	66.7	42	7.1	0	
	NGBUNGBU	129	81.4	7	42.9	122	18.9	0		25	0	0	
	NYODU	0		0		683	5.4	4	0	267	0.7	0	
	NZURU	12	100	0		113	7.1	0		54	5.6	0	
	PALEY	0		0		42	0	0		3	0	0	
	PAMUNDU	3	0	0		0		0		0		0	
	RAA	2	0	0		0		0		0		0	
	RUJU	132	87.9	12	83.3	149	22.8	1	0	69	5.8	0	
	Thecer	0		0		13	0	0		1	0	0	
	THEDÉJÀ 1	0		0		90	1.1	0		16	0	0	
	THEDEJA 2	0		0		105	2.9	0		26	0	0	
	UCUDO	0		0		40	7.5	0		4	0	0	
	ULYEKO	12	25.0	2	50.0	0		0		0		0	
	UMULO	314	73.2	10	70.0	254	12.2	0		70	0	0	
	URYANG	0		0		75	6.7	0		10	0	0	
	VUNA	0		0		27	7.4	0		2	0	0	

		2010	2010	2010	2010	2021-2023	2021-2023	2021-2023	2021-2023	2021-2023	2021-2023	2021-2023	2021-2023
**Age group**		**≥18**	**≥18**	**12-17**	**12-17**	**≥18**	**≥18**	**≥18**	**≥18**	**12-17**	**12-17**	**12-17**	**12-17**
**Prior IVM Tx**		**No**	**No**	**No**	**No**	**No**	**No**	**Yes**	**Yes**	**No**	**No**	**Yes**	**Yes**
**Zone de Santé**	**Village**	**N**	**% >0**	**N**	**% >0**	**N**	**% >0**	**N**	**% >0**	**N**	**% >0**	**N**	**% >0**
	WI RAA	0		0		59	3.4	0		15	0	0	
	WILOO	0		0		45	4.4	0		5	0	0	
	YAU	63	61.9	4	75.0	462	8.7	0		211	1.4	0	
Nyarambe	Awora	4	100	0		0		0		0		0	
	Gbii	0		0		36	11.1	0		20	0	0	
	Jupafoyo	0		0		84	0	0		53	0	0	
	Jupajalbonyo	0		0		37	0	3	0	3	0	0	
	Jupanyamoro	0		0		122	4.1	0		76	0	0	
	jupasugu	0		0		102	3.9	0		57	0	0	
	Jupawalu	0		0		68	0	0		53	0	0	
	Jupawegi	0		0		3	0	1	0	0		0	
	Jupudero 1	0		0		61	3.3	0		31	0	0	
	jupudero 2	21	76.2	0		14	0	2	0	3	0	0	
	Jupujanga	0		0		29	10.3	0		8	0	0	
	Jupuvuga	0		0		99	10.1	0		45	0	0	
	Kpanyi	1	0	0		35	2.9	3	0	8	0	0	
	Mbraze	1	100	0		0		0		0		0	
	Pacung	2	100	0		0		0		0		0	
	Umulo 1	7	42.9	0		0		0		0		0	

References

1. Opoku NO, Bakajika DK, Kanza EM, Howard H, Mambandu GL, Nyathirombo A, et al. Single dose moxidectin versus ivermectin for Onchocerca volvulus infection in Ghana, Liberia, and the Democratic Republic of the Congo: a randomised, controlled, double-blind phase 3 trial. Lancet. 2018;392:1207-16. doi: S0140-6736(17)32844-1 [pii];10.1016/S0140-6736(17)32844-1 [doi].
2. Bakajika D, Kanza EM, Opoku NO, Howard HM, Mambandu GL, Nyathirombo A, et al. Effect of a single dose of 8 mg moxidectin or 150 mug/kg ivermectin on O. volvulus skin microfilariae in a randomized trial: Differences between areas in the Democratic Republic of the Congo, Liberia and Ghana and impact of intensity of infection. PLoS Negl Trop Dis. 2022;16(4):e0010079. doi: 10.1371/journal.pntd.0010079 [doi];PNTD-D-21-01732 [pii].
3. Kanza EM, Nyathirombo A, Larbelee JP, Opoku NO, Bakajika DK, Howard HM, et al. Onchocerca volvulus microfilariae in the anterior chambers of the eye and ocular adverse events after a single dose of 8 mg moxidectin or 150 µg/kg ivermectin: results of a randomized double-blind Phase 3 trial in the Democratic Republic of the Congo, Ghana and Liberia. Parasit Vectors. 2024;17(1):137. doi: 10.1186/s13071-023-06087-3.
4. Kura K, Milton P, Hamley JID, Walker M, Bakajika DK, Kanza EM, et al. Can mass drug administration of moxidectin accelerate onchocerciasis elimination in Africa? Philos Trans R Soc Lond B Biol Sci. 2023;378(1887):20220277. doi: 10.1098/rstb.2022.0277.
5. Bakajika DK, Nigo MM, Lotsima JP, Masikini GA, Fischer K, Lloyd MM, et al. Filarial antigenemia and Loa loa night blood microfilaremia in an area without bancroftian filariasis in the Democratic Republic of Congo. Am J Trop Med Hyg. 2014;91(6):1142-8. doi: ajtmh.14-0358 [pii];10.4269/ajtmh.14-0358 [doi].
6. Dusabimana A, Bhwana D, Mandro M, Mmbando BP, Siewe Fodjo JN, Colebunders R. OV16 Seroprevalence among Persons with Epilepsy in Onchocerciasis Endemic Regions: A Multi-Country Study. Pathogens. 2020;9(10). doi: pathogens9100847 [pii];10.3390/pathogens9100847 [doi].
7. Dusabimana A, Bhwana D, Raimon S, Mmbando BP, Hotterbeekx A, Tepage F, et al. Ivermectin Treatment Response in Onchocerca Volvulus Infected Persons with Epilepsy: A Three-Country Short Cohort Study. Pathogens. 2020;9(8). doi: pathogens9080617 [pii];10.3390/pathogens9080617 [doi].
8. Dusabimana A, Mandro MN, Siewe Fodjo JN, Dolo H, Coenen S, Colebunders R. Community perceptions and attitudes regarding epilepsy and disease cost after implementation of a community-based epilepsy treatment program in onchocerciasis-endemic communities in the Democratic Republic of Congo. Epilepsy Behav. 2021;116:107773. doi: S1525-5050(21)00007-X [pii];10.1016/j.yebeh.2021.107773 [doi].
9. Dusabimana A, Tsebeni WS, Raimon SJ, Fodjo JNS, Bhwana D, Tepage F, et al. Effect of Ivermectin Treatment on the Frequency of Seizures in Persons with Epilepsy Infected with Onchocerca volvulus. Pathogens. 2020;10(1). doi: pathogens10010021 [pii];10.3390/pathogens10010021 [doi].
10. Hotterbeekx A, Dusabimana A, Mandro M, Abhafule GM, Deogratias W, Siewe Fodjo JN, et al. Urinary N-acetyltyramine-O,beta-glucuronide in Persons with Onchocerciasis-Associated Epilepsy. Pathogens. 2020;9(3). doi: pathogens9030191 [pii];10.3390/pathogens9030191 [doi].
11. Hotterbeekx A, Perneel J, Mandro M, Abhafule G, Siewe Fodjo JN, Dusabimana A, et al. Comparison of Diagnostic Tests for Onchocerca volvulus in the Democratic Republic of Congo. Pathogens. 2020;9(6). doi: pathogens9060435 [pii];10.3390/pathogens9060435 [doi].
12. Hotterbeekx A, Vieri MK, Ramberger M, Jozefzoon-Aghai A, Mandro M, Tepage F, et al. No Evidence for the Involvement of Leiomodin-1 Antibodies in the Pathogenesis of

Onchocerciasis-Associated Epilepsy. Pathogens. 2021;10(7). doi: pathogens10070845 [pii];10.3390/pathogens10070845 [doi].
13. Mandro M, Siewe Fodjo JN, Dusabimana A, Mukendi D, Haesendonckx S, Lokonda R, et al. Single versus Multiple Dose Ivermectin Regimen in Onchocerciasis-Infected Persons with Epilepsy Treated with Phenobarbital: A Randomized Clinical Trial in the Democratic Republic of Congo. Pathogens. 2020;9(3). doi: pathogens9030205 [pii];10.3390/pathogens9030205 [doi].
14. Mandro M, Siewe Fodjo JN, Mukendi D, Dusabimana A, Menon S, Haesendonckx S, et al. Ivermectin as an adjuvant to anti-epileptic treatment in persons with onchocerciasis-associated epilepsy: A randomized proof-of-concept clinical trial. PLoS Negl Trop Dis. 2020;14(1):e0007966. doi: 10.1371/journal.pntd.0007966 [doi];PNTD-D-19-01258 [pii].
15. Raimon S, Dusabimana A, Abd-Elfarag G, Okaro S, Carter JY, Newton CR, et al. High Prevalence of Epilepsy in an Onchocerciasis-Endemic Area in Mvolo County, South Sudan: A Door-To-Door Survey. Pathogens. 2021;10(5). doi: pathogens10050599 [pii];10.3390/pathogens10050599 [doi].
16. Vieri MK, Hotterbeekx A, Mandro M, Siewe Fodjo JN, Dusabimana A, Nyisi F, et al. Serotonin Levels in the Serum of Persons with Onchocerciasis-Associated Epilepsy: A Case-Control Study. Pathogens. 2021;10(6). doi: pathogens10060720 [pii];10.3390/pathogens10060720 [doi].
17. Post RJ, Laudisoit A, Mandro M, Lakwo T, Laemmer C, Pfarr K, et al. Identification of the onchocerciasis vector in the Kakoi-Koda focus of the Democratic Republic of Congo. PLoS Negl Trop Dis. 2022;16(11):e0010684. doi: 10.1371/journal.pntd.0010684.
18. Hedtke SM, Kode A, Ukety TO, Mande JL, Abhafule GM, Raciu AA, et al. Procedure for Handling and Storage of Onchocerca volvulus Microfilariae Obtained from Skin Snips for Downstream Genetic Work. Trop Med Infect Dis. 2023;8(9). doi: 10.3390/tropicalmed8090445.
19. Lakwo T, Ukety T, Bakajika D, Tukahebwa E, Awaca P, Amazigo U. "Cross-border collaboration in onchocerciasis elimination in Uganda: progress, challenges and opportunities from 2008 to 2013". Global Health. 2018;14(1):16. doi: 10.1186/s12992-018-0333-1 [doi];10.1186/s12992-018-0333-1 [pii].
20. Mandro Ndahura M. Epilepsy in onchocerciasis endemic villages of Democratic Republic of Congo : epidemiology, clinical and treatment aspects. University of Antwerp, Faculty of Medicine & Health sciences; 2020. https://repository.uantwerpen.be/docstore/d:irua:3109, accessed 14 July 2025
21. Nigo MM. Schistosomiasis in Eastern Democratic Republic of the Congo: A major neglected healthcare concern. Basel: University Basel Faculty for Medicine; 2020 https://edoc.unibas.ch/entities/publication/5cec52a5-9cf6-4362-8dff-3eb1a245acea, accessed 14 July 2025
22. Coffeng LE, Pion SD, O'Hanlon S, Cousens S, Abiose AO, Fischer PU, et al. Onchocerciasis: the pre-control association between prevalence of palpable nodules and skin microfilariae. PLoS Negl Trop Dis. 2013;7(4):e2168. doi: 10.1371/journal.pntd.0002168 [doi];PNTD-D-12-00825 [pii].
23. Mandro M, Suykerbuyk P, Tepage F, Rossy D, Ngave F, Hasan MN, et al. Onchocerca volvulus as a risk factor for developing epilepsy in onchocerciasis endemic regions in the Democratic Republic of Congo: a case control study. Infect Dis Poverty. 2018;7(1):79. doi: 10.1186/s40249-018-0465-9.
24. Lenaerts E, Mandro M, Mukendi D, Suykerbuyk P, Dolo H, Wonya'Rossi D, et al. High prevalence of epilepsy in onchocerciasis endemic health areas in Democratic Republic of the Congo. Infect Dis Poverty. 2018;7(1):68. doi: 10.1186/s40249-018-0452-1.

Abbreviations

AdS	Health area(s) (Aire(s) de Santé, lowest health system administrative unit)
APOC	African Programme for Onchocerciasis Control

CDTI	Community directed treatment with ivermectin
CI	95% Confidence interval
DRC	Democratic Republic of the Congo
G-FDA	Food and Drugs Authority of Ghana
MDAi	Mass drug administration of ivermectin
MDGH	Medicines Development for Global Health
mf	Microfilaria
MoH	Ministère de la Santé Publique, Hygiène et Prevention of the Democratic Republic of the Congo
OR	Odds ratio
REA	Rapid Epidemiological Assessment
SmfD	Skin microfilariae density (mf/mg skin)
SSA	Sub-Saharan Africa
US-FDA	Food and Drug Administration of the United States of America
WHO	World Health Organization
WHO/ESPEN	WHO Expanded Special Project for Elimination of Neglected Tropical Diseases
WHO/TDR	UNICEF/UNDP/World Bank/WHO Special Programme for Research and Training in Tropical Diseases
ZdS	Health Zone(s) (Zone(s) de Santé)
